# Kynurenine pathway metabolites in cerebrospinal fluid and blood as potential biomarkers in Huntington's disease

**DOI:** 10.1111/jnc.15360

**Published:** 2021-05-05

**Authors:** Filipe B. Rodrigues, Lauren M. Byrne, Alexander J. Lowe, Rosanna Tortelli, Mariette Heins, Gunnar Flik, Eileanoir B. Johnson, Enrico De Vita, Rachael I. Scahill, Flaviano Giorgini, Edward J. Wild

**Affiliations:** ^1^ UCL Huntington's Disease Centre UCL Queen Square Institute of Neurology University College London London UK; ^2^ Charles River Laboratories Groningen The Netherlands; ^3^ Lysholm Department of Neuroradiology National Hospital for Neurology & Neurosurgery London UK; ^4^ Department of Biomedical Engineering School of Biomedical Engineering and Imaging Sciences King's College London London UK; ^5^ Department of Genetics and Genome Biology University of Leicester Leicester UK

**Keywords:** biomarkers, blood, cerebrospinal fluid, cohort studies, Huntington's disease, Kynurenine

## Abstract

Converging lines of evidence from several models, and post‐mortem human brain tissue studies, support the involvement of the kynurenine pathway (KP) in Huntington's disease (HD) pathogenesis. Quantifying KP metabolites in HD biofluids is desirable, both to study pathobiology and as a potential source of biomarkers to quantify pathway dysfunction and evaluate the biochemical impact of therapeutic interventions targeting its components. In a prospective single‐site controlled cohort study with standardised collection of cerebrospinal fluid (CSF), blood, phenotypic and imaging data, we used high‐performance liquid‐chromatography to measure the levels of KP metabolites—tryptophan, kynurenine, kynurenic acid, 3‐hydroxykynurenine, anthranilic acid and quinolinic acid—in CSF and plasma of 80 participants (20 healthy controls, 20 premanifest HD and 40 manifest HD). We investigated short‐term stability, intergroup differences, associations with clinical and imaging measures and derived sample‐size calculation for future studies. Overall, KP metabolites in CSF and plasma were stable over 6 weeks, displayed no significant group differences and were not associated with clinical or imaging measures. We conclude that the studied metabolites are readily and reliably quantifiable in both biofluids in controls and HD gene expansion carriers. However, we found little evidence to support a substantial derangement of the KP in HD, at least to the extent that it is reflected by the levels of the metabolites in patient‐derived biofluids.

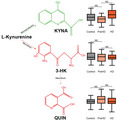

## Graphical abstract

Converging lines of evidence from non‐human models support the involvement of the kynurenine pathway in Huntington's disease. However, evidence in living humans is limited. In a prospective study with standardised collection of cerebrospinal fluid and blood, we used high‐performance liquid‐chromatography to analyse this pathway. We concluded that the selected metabolites are readily and reliably quantifiable. Yet, we found little evidence to support a substantial derangement of the kynurenine pathway in Huntington's disease, at least to the extent that it is reflected by the levels of the metabolites in patient‐derived biofluids. 3‐HK, 3‐hydroxykynurenine; KYNA, kynurenic acid; QUIN, quinolinic acid.

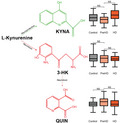

**Abbreviations**
3‐HANA: 3‐hydroxyanthranilic acid
3‐HK: 3‐hydroxykynurenine
95% CI: 95% confidence interval
AA: anthranilic acid
CAG: cytosine‐adenine‐guanine
CSF: cerebrospinal fluid
cUHDRS: Composite Unified Huntington's Disease Rating Scale
DBS: disease burden score
DCL: UHDRS diagnostic confidence level
GRAPPA: GeneRalized autocalibrating partial parallel acquisition
HD: huntington's disease
HPLC: high‐performance liquid‐chromatography
HTRF: homogeneous time‐resolved Förster resonance energy transfer assay
*HTT*
: huntingtin gene
ICC: interclass correlation coefficient
IDO1/2: indoleamine‐2,3‐dioxygenase 1/2
KATs: kynurenine aminotransferases
KMO: kynurenine 3‐monooxygenase
KP: kynurenine pathway
KYN: kynurenine
KYNA: kynurenic acid
LC‐MC: HPLC with MS/MS detection method
LoQ: limit of quantification
MALP‐EM: multi‐atlas propagation with EM refinement
mHTT: mutant huntingtin protein
MIDAS: medical image display analysis software
MRI: magnetic resonance imaging
MRM: multiple‐reaction‐monitoring
MS/MS: tandem mass spectroscopy
MSN: medium spiny neuron
NAD+: Nicotinamide adenine dinucleotide
NMDA: *N*‐methyl‐D‐aspartate receptors
PreHD: premanifest Huntington's disease
QCs: quality control samples
QUIN: quinolinic acid
RRID: research resource identifier (see scicrunch.org)
SCN: stroop color naming
SDMT: symbol digit modalities test
SWR: stroop word reading
TDO: tryptophan‐2,3‐dioxygenase
TFC: UHDRS total functional capacity
TIV: total intracranial volume
TMS: UHDRS total motor score
TRP: tryptophan
UHDRS: unified Huntington's disease rating scale
VFC: verbal fluency categorical

## 1 INTRODUCTION

Huntington's disease (HD) is an invariably fatal neurodegenerative disease caused by CAG repeat expansions in the *HTT* gene. Inherited in an autosomal dominant manner, the polyglutamine expansion results in the ubiquitous expression of a mutant form of huntingtin protein (mHTT) (McColgan & Tabrizi, 2018), causing a diverse array of intracellular toxicities and derangement of downstream pathways. With no treatments shown to prevent, slow or reverse its progression (Travessa et al., 2017), extensive dysfunction and neuronal death occurs. Although characterized by the degeneration of striatal medium spiny neurons (MSNs), widespread damage involving most brain regions is observed (Bates et al., 2015). In addition to its expression in glial cells and neurons, mHTT is expressed in the peripheral nervous system (van der Burg et al., 2009). The interplay amongst different cell types, both central and peripheral, and the dynamics of dysfunction versus compensation are increasingly recognised as contributing to the complex pathogenesis of HD (Carroll et al., 2015; Gregory et al., 2018; Wood et al., 2018).

Early animal models used excitotoxins such as quinolinic acid (QUIN), injected into the striatum, to recapitulate specific cellular pathology and phenotypic features of HD (Beal et al., 1986; Schwarcz et al., 1983). Although influential, chemically lesioned models have now largely been superseded by various transgenic animals (Yang & Chan, 2011). The observed selective vulnerability of striatal MSNs to such toxins led to the theory that excitotoxicity may be an inherent part of HD pathogenesis, a theory that remains of interest.

**FIGURE 1:**
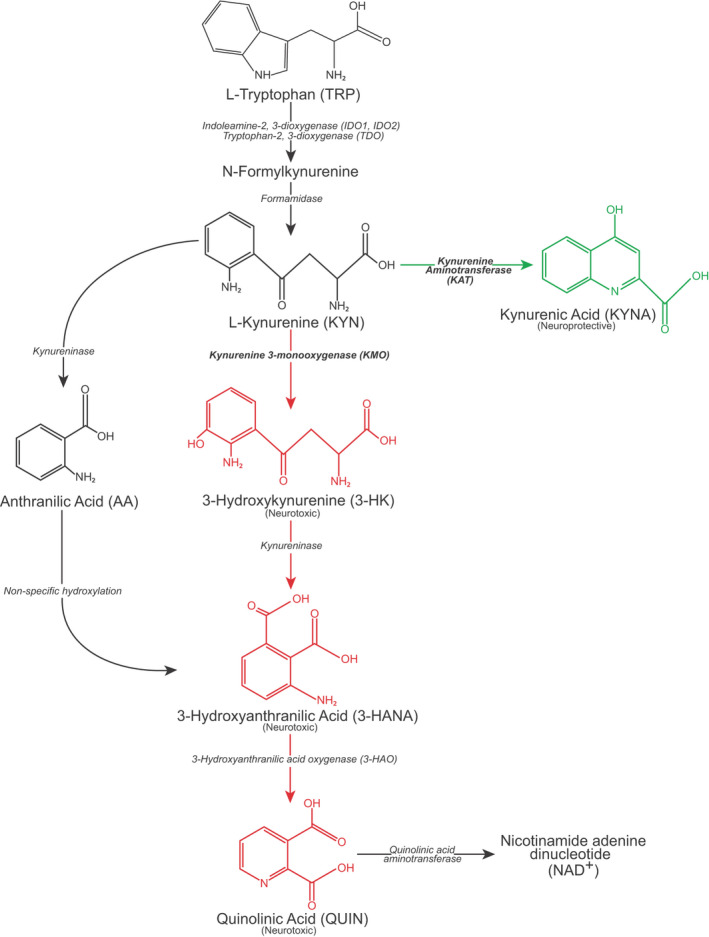
Overview of the kynurenine pathway. Adapted with authors' and editors' permission (Maddison & Giorgini, 2015). The designations ‘neurotoxic’ and ‘neuroprotective’ are assigned on the basis of the balance of evidence and are acknowledged to be simplifications of complex properties

QUIN is predominantly produced in the central nervous system by microglial cells, as one endpoint of the kynurenine pathway (KP) of tryptophan (TRP) degradation (Figure 1) (Maddison & Giorgini, 2015), ultimately leading to the formation of the coenzyme nicotinamide adenine dinucleotide (NAD+). In addition to promoting excitotoxicity via activation of *N*‐methyl‐D‐aspartate (NMDA) receptors, QUIN is a potent free radical generator (Rios & Santamaria, 1991). Several additional KP metabolites have been found to be neuroactive, including 3‐hydroxykynurenine (3‐HK) and kynurenic acid (KYNA). 3‐HK is broadly neurotoxic via generation of free radicals (Colin‐Gonzalez et al., 2013; Hiraku et al., 1995; Ishii et al., 1992), whereas KYNA displays neuroprotective properties via antagonism of excitatory amino acid receptors and scavenging of free radicals (Carpenedo et al., 2001; Foster et al., 1984; Goda et al., 1999; Vecsei & Beal, 1990). The absolute levels of these metabolites, as well as the ratio of the neurotoxic compounds relative to KYNA, may be of value when considering progression of neurodegenerative disorders such as HD. Furthermore, the activities of the key KP regulatory enzymes kynurenine 3‐monooxygenase (KMO)—which synthesizes 3‐HK leading to downstream formation of QUIN—and the kynurenine aminotransferases (KATs) which synthesize KYNA—are equally important (Maddison & Giorgini, 2015).

Imbalances in KP metabolites have been associated with several neurodegenerative disorders, (e.g., Alzheimer's and Parkinson's disease), psychiatric disorders (e.g., schizophrenia, bipolar disorder), as well as cancer and autoimmune diseases (Åkesson et al., 2018; Cao et al., 2021; Sorgdrager et al., 2019; Sumitomo et al., 2021; Trepci et al., 2021). Converging lines of evidence support the involvement of the KP in HD pathogenesis. Although the mechanisms are not fully understood, immune activation and transcriptional dysregulation are among the more likely candidates. In post‐mortem brain tissue obtained from HD patients, QUIN and 3‐HK levels are increased and KYNA levels reduced (Beal et al., 1992; Guidetti et al., 2006; Sathyasaikumar et al., 2018). In the R6/2 mouse model, KMO activity has shown to be increased (Sathyasaikumar et al., 2010); however, recent work in an HD patient cohort failed to replicate these findings (Vonsattel neuropathology grades 1 through 4) (Sathyasaikumar et al., 2018). Furthermore, inhibition of KMO has demonstrated beneficial effects in multiple studies. In HD mouse models, peripheral KMO inhibition improves disease‐relevant phenotypes (Beaumont et al., 2016; Zwilling et al., 2011), while genetic inhibition is strongly protective in HD model yeast (Giorgini et al., 2005) and *Drosophila* (Breda et al., 2016; Campesan et al., 2011; Green et al., 2012). Consequently, pharmacological inhibition of KMO is considered a promising target for HD therapeutic development (Maddison & Giorgini, 2015; Zhang et al., 2019).

Evidence of KP involvement in living human HD patients is limited. Blood and cerebrospinal fluid (CSF) are accessible biofluids that offer insights into comparative derangements in the periphery and CNS and are sources of useful biomarkers in HD (Byrne et al., 2017a; Byrne et al., 2018b; Byrne & Wild, 2016); however, evidence on the KP in these fluids in HD is limited and conflicting. Early work examining CSF identified no difference in CSF QUIN levels in 10 HD patients versus 7 controls with schizophrenia (Schwarcz et al., 1988). This was further supported by Heyes et al. who observed no significant differences in CSF QUIN in 9 HD patients compared to 9 hospital patients using electron capture negative chemical ionization mass spectrometry and gas chromatography (Heyes et al., 1991). In contrast with QUIN, levels of CSF kynurenine (KYN) and KYNA were shown to be mildly reduced in 13 HD patients compared to 7 healthy controls (Heyes et al., 1992).

Notably, all previous published human studies of the KP in HD CSF pre‐date the discovery of the causative genetic mutation, used small sample numbers and did not control for important sources of variability like time of day, fasting status, CSF processing methods and source of control CSF (Rodrigues et al., 2018).

Quantifying KP metabolites in HD CSF remains desirable, both to study pathobiology in living human patients, and as a potential source of biomarkers to quantify pathway dysfunction and the biochemical impact of therapeutic interventions targeting its components. Therefore, we sought to combine modern analytical methods, with CSF and matched blood plasma, collected and processed under strictly standardised conditions, from a large (*n* = 80) prospective cohort of gene expansion carriers and matched controls (Byrne et al., 2017a; Rodrigues et al., 2020). Using high‐performance liquid‐chromatography (HPLC) with tandem mass spectroscopy (MS/MS) quantification methods, we measured levels of six KP metabolites (Figure 1)—TRP, L‐kynurenine (L‐KYN), KYNA, 3‐HK, anthranilic acid (AA) and QUIN—in CSF and blood plasma, and applied a predefined statistical analysis plan to investigate the hypothesis that KMO activity is important to HD pathogenesis. As a predefined secondary analysis, we examined ratios of metabolites that may be informative of the activity of key enzymes in the pathway. Finally, we undertook an exploratory analysis of all metabolites in CSF and plasma in HD and controls.

## 2 MATERIALS AND METHODS

This article was posted on medRxiv on 6th August 2020 (https://doi.org/10.1101/2020.08.06.20169524) (Rodrigues et al., 2020).

### 2.1 Study design

The HD‐CSF study was a prospective single‐site controlled cohort study with standardised collection of CSF, blood and phenotypic data (preregistered with the UK Medical Research Council and NHS Health Research Authority; online protocol: https://doi.org/10.5522/04/11828448.v1). Recruitment figures were based on a priori sample size calculations to detect cross‐sectional differences in CSF mHTT—the primary outcome measure of the HD‐CSF study—between healthy controls and gene expansion carriers, and to investigate longitudinal changes over 2 years, based on effect sizes from previous reports (Wild et al., 2015); but the collection was also designed to generate a collection of samples and matched data for other exploratory and validatory biomarker analyses. Analysing the KP was a prespecified intention for the HD‐CSF study. Eighty participants were recruited (20 healthy controls, 20 premanifest HD [PreHD], and 40 manifest HD). All phenotypic assessment measures were predefined for HD‐CSF based on metrics shown to have the largest effect sizes for predicting HD progression (Tabrizi et al., 2013). Baseline assessments were conducted from February 2016 to February 2017 (Byrne et al., 2018b). At baseline, 15 (19%) participants underwent an optional repeat sampling 4–8 weeks after baseline, permitting the assessment of within‐subject short‐term metabolite stability. MRI brain imaging, an optional component, was completed by 64 participants (80%). Clinical assessments were not blind to group membership, although biofluid and imaging measurements and statistical analysis were.

### 2.2 Ethical approval

This study was performed in accordance with the principles of the Declaration of Helsinki, and the International Conference on Harmonization Good Clinical Practice standards. Ethical approval was obtained from the London Camberwell St Giles Research Ethics Committee (15/LO/1917). Prior to undertaking study procedures, all participants gave informed consent which was obtained by clinical staff.

### 2.3 Participants

All potentially eligible participants were consecutively approached in the National Hospital for Neurology & Neurosurgery Huntington's Disease Multidisciplinary Clinic or via the University College London Huntington's Disease Centre research databases, until the study was fully recruited. Manifest HD participants were defined as adults having a Unified Huntington's Disease Rating Scale (UHDRS) diagnostic confidence level (DCL) of 4 and *HTT* CAG repeat count ≥ 36. PreHD participants had CAG ≥ 40 and DCL < 4. Healthy controls were age‐ and gender‐matched to gene expansion carriers, mostly not at‐risk spouses (i.e., without family history) or gene‐negative siblings of HD gene expansion carriers and with no neurological signs or symptoms. Other inclusion criteria were: 18–75 years of age, inclusive; capacity to provide consent and comply with study procedures; Enroll‐HD study (NCT01574053) participation. Exclusion criteria included: concurrent participation in a clinical trial; alcohol or recreational drug intoxication or abuse; inappropriate or unstable use of medications to treat HD symptoms in the previous 30 days; significant medical, neurological or psychiatric co‐morbidity likely interfere with sample quality or safety of study procedures as assessed by the medical history and physical exam; needle phobia, frequent headaches, significant lower spine deformity or major surgery; antiplatelet or anticoagulant use in the previous 14 days; abnormal full blood count, coagulation parameters or C‐reactive protein >2× the upper limit of normal.

### 2.4 Clinical assessments

Motor, cognitive and functional status were assessed using the UHDRS from the core Enroll‐HD battery (Landwehrmeyer et al., 2017), including the UHDRS Total Motor Score (TMS), Total Functional Capacity (TFC), Symbol Digit Modalities Test (SDMT), Stroop Word Reading (SWR), Stroop Color Naming (SCN) and Verbal Fluency—Categorical (VFC). These were performed at either a screening visit before sampling or an associated Enroll‐HD visit (https://www.enroll‐hd.org) within the 2 months prior to screening. We employed a calibrated iteration of the composite UHDRS (cUHDRS) (Schobel et al., 2017; Trundell et al., 2018). Disease burden score (DBS) was calculated for each gene expansion carrier using the formula: [CAG – 35.5] × age (Penney et al., 1997). DBS estimates cumulative HD pathology as a function of CAG and the time exposed to the effects of the pathologic mutation and has been shown to predict several features of disease progression including striatal pathology (Penney et al., 1997; Tabrizi et al., 2013).

### 2.5 Biosample collection and processing

CSF and matched plasma were obtained as previous described (Byrne et al., 2018b). All collections were standardised for time of day after overnight fasting and processed within 30 min of collection using standardised equipment. A lumbar puncture was performed using a 22G Whitacre atraumatic BD® spinal needle. Up to 20 ml of CSF were collected into a 50 ml precooled polypropylene collection tube on wet ice. Blood was collected within 10 min of CSF into 10 ml lithium heparin BD® tubes and placed on wet ice. Biosamples were transported to the laboratory on wet ice, and sample processing started within 15 min of collection. CSF was centrifuged at 400 *g* for 10 min at 4°C to remove cells and aliquoted into 300 µl cryovials. Blood was centrifuged at 1,300 *g* for 10 min at 4°C and the supernatant was aliquoted into 300 µl cryovials. All surfaces in contact with the biosamples were polypropylene. Biosamples were frozen and stored at −80°C until quantification.

### 2.6 Kynurenine pathway metabolite quantification

KP metabolites were quantified by Charles River Laboratories (the Netherlands) using a high‐performance liquid‐chromatography (HPLC) with tandem mass spectroscopy (MS/MS) detection method (LC‐MC) published previously (Beaumont et al., 2016). D_5_‐TRP, D_4_‐KYN, D_5_‐KYNA, ^13^C_6_‐3‐HK, D_4_‐AA and a D_3_‐QUIN were used as internal standards. An aliquot of a solution containing the internal standard was mixed with an aliquot of each experimental sample to generate an LC‐MS sample. An aliquot of each LC‐MS sample was injected into the HPLC system by an automated sample injector (SIL20‐AD, Shimadzu, Japan). Chromatographic separation was performed using a reversed phase analytical column, configured as per Table 1, with elution performed using a linear gradient. MS analyses were performed using an API 4,000 MS/MS system consisting of an API 4,000 MS/MS detector and a Turbo Ion Spray interface (Applied Biosystems, USA). The acquisitions on API 4,000 were performed in positive ionization mode, with optimized settings for the analytes. The instrument was operated in multiple‐reaction‐monitoring (MRM) mode. Data were calibrated and quantified using the Analyst™ data system (Applied Biosystems, USA).

**TABLE 1:** Chromatographic separation parameters for each metabolite and assay performance

Metabolite	Column size (mm)	Particle size (µm)	Temperature (°C)	Mobile phase A	Mobile phase B	Elution flow rate (ml/min)	Low limit of quantification (LoQ)	*n* (%) below LoQ	*n* (%) quality rejected
TRP	100 × 3	2.5	5	H_2_O + 0.1% FA	ACN + 0.1% FA	0.3	C: 0.5 μM P: 0.5 μM	C: 0/95 (0%) P: 0/93 (0%)	C: 0/95 (0%) P: 0/93 (0%)
KYN	150 × 2.1	3	25	H_2_O + 0.1% FA	ACN + 0.1% FA	0.2	C: 2.5 nM P: 0.5 μM	C: 0/95 (0%) P: 0/93 (0%)	C: 0/95 (0%) P: 0/93 (0%)
KYNA	150 × 2.1	3	25	H_2_O + 0.1% FA	ACN + 0.1% FA	0.2	C: 0.25 nM P: 15.0 nM	C: 0/95 (0%) P: 4/93 (4%)	C: 0/95 (0%) P: 4/93 (4%)
3‐HK	150 × 2.1	3	25	H_2_O + 0.1% FA	ACN + 0.1% FA	0.2	C: 0.25 nM P: 5.0 nM	C: 0/95 (0%) P: 1/93 (1%)	C: 0/95 (0%) P: 0/93 (0%)
AA	150 × 2.1	3	25	H_2_O + 0.1% FA	ACN + 0.1% FA	0.2	C: 1.25 nM P: 5.0 nM	C: 0/95 (0%) P: 7/93 (8%)	C: 0/95 (0%) P: 0/93 (0%)
QUIN	100 × 3	2.5	35	H_2_O + 0.2% TFA	ACN + 0.2% TFA	0.3	C: 1.0 nM P: 100 nM	C: 0/95 (0%) P: 1/93 (1%)	C: 0/95 (0%) P: 0/93 (0%)

Note

Note that 2 manifest HD participants are missing plasma.

3‐HK, 3‐hydroxykynurenine; AA, anthranilic acid; acetonitrile; ACN; C, cerebrospinal fluid; FA, formic acid; H_2_O, ultra‐purified water; KYN, kynurenine; KYNA, kynurenic acid; P, plasma; QUIN, quinolinic acid; TFA, trifluoroacetic acid; TRP, tryptophan.

Assays were performed blinded to clinical data and in a continuous run with the same quality control samples (QCs) to confirm performance over time. Each sample was measured once. The acceptance criteria for CSF assays was ±25% accuracy for the low limits of quantification (LoQ) calibrator and QC‐low, and ±20% accuracy for all other calibrators and QC‐mid and ‐high. For plasma assays, acceptance criteria were 5% wider. Individual runs were accepted when >66% of QC's were within criteria as described above (>50% on individual level). The LoQ, frequency of samples below LoQ, and frequency of rejected samples due to quality criteria are shown in Table 1.

### 2.7 MRI acquisition

T1‐weighted MRI data were acquired on a 3T Siemens Prisma scanner using a protocol optimized for this study. Images were acquired using a 3D magnetization‐prepared radio‐frequency pulses and rapid gradient‐echo (MPRAGE) sequence with 2 s repetition time, 2.05 ms echo time, 850 ms inversion time, flip angle of 8 degrees, and matrix size 256 × 240 mm; 256 coronal partitions were collected to cover the entire brain with a slice thickness of 1 mm. Parallel imaging acceleration (GeneRalized Autocalibrating Partial Parallel Acquisition [GRAPPA], acceleration factor [R] = 2) was used and 3D distortion correction was applied to all images.

### 2.8 MRI processing

All T1‐weighted scans passed visual quality control check for the presence of significant motion or other artefacts before processing. Bias correction was performed using the N3 procedure (Sled et al., 1998). A semiautomated segmentation procedure via Medical Image Display Analysis Software (MIDAS) was used to generate volumetric regions of the whole brain and total intracranial volume (TIV), as previously described (Freeborough et al., 1997; Scahill et al., 2003; Whitwell et al., 2001). SPM12 ‘Segment’ (MATLAB version 2012) was used to measure the volume of the grey and white matter (Ashburner & Friston, 2000). Multi‐Atlas Propagation with EM Refinement (MALP‐EM) was used to quantify caudate volume (Ledig et al., 2015). MALP‐EM is an automated tool used to segment MRI scans into regional volumes and has previously been validated for use in HD cohorts (Johnson et al., 2017). Default settings were used for both SPM12 segmentations and MALP‐EM caudate regions. No scans failed processing after visual quality control of segmentations by experienced raters to ensure accurate delineation of the regions. Baseline MRI volumes were presented adjusted for TIV. All MRI analyses used brain volumes as percentage of TIV.

### 2.9 Statistical analyses

Statistical analysis was performed with Stata MP 16 software (RRID:SCR_012763; StataCorp, USA).

A priori sample size calculations for two 2‐group comparisons (i.e., healthy control vs. PreHD and PreHD vs. manifest HD) using 2‐sided Student *t* tests, assuming a 5% type 1 error (i.e., rejecting a true null hypothesis) rate, 20% type 2 error (i.e., not rejecting a false null hypothesis; 80% statistical power) rate, and adapting effect sizes reported in Heyes et al., (1992) for KYNA and QUIN, resulted in samples with less than 5 participants per group when accounting for 2 comparisons and 10 independent hypotheses. Using the same assumptions and a smaller effect size (i.e., half of reported by Heyes et al., 1992), resulted in samples with less than 10 participants per group for a similar study design.

To minimise type 1 errors (i.e., rejection of true null hypotheses) resulting from multiplicity, we predefined CSF levels of KYNA, 3‐HK and QUIN as primary outcomes, hypothesising that quantifiable differences would be apparent if the KP is dysregulated in HD. The ratios of CSF 3‐HK:KYNA, KYNA:KYN and 3‐HK:KYN were secondary outcomes, hypothesised to reflect the overall balance of the key neuroprotective and neurotoxic metabolites, the activity of the KATs, and the activity of KMO respectively (Figure 1). Analyses of all other CSF and plasma metabolites were exploratory. At the helpful suggestion of a peer‐reviewer, we also added the CSF KYN:TRP ratio as a post hoc exploratory analysis. This ratio reflects the activity of the indoleamine‐2,3‐dioxygenases (IDO1 and IDO2) and tryptophan‐2,3‐dioxygenase (TDO) enzymes.

Measurements below the LoD for the metabolites of interest were assumed as missing—no imputation was used. Analyte distributions were visually assessed and if necessary, arithmetical transformations were applied to meet model assumptions. We provide a graphical depiction of the distributions before and after transformations (Figures S1–S3 and S10) and a figure showing the effects of each transformation (Figure S4). Of note that for outcomes where a transformation includes a reciprocal (i.e., 1/x), an increase in the raw measurement corresponds to a decreased in the transformed value and vice‐versa. Continuous variables were reported as mean ± standard deviations (*SD*); and categorical variables as absolute (*n*) and relative frequencies (%).

Associations with age (healthy controls only), gender, CAG repeat count (gene expansion carriers only) and haemoglobin (in case of CSF metabolites only) and time in freezer storage were visually assessed for all outcomes (Figures S5 and S6). Due to its known effects on HD, all models included age as covariate.

As a planned second‐level analysis, to assess associations with measures beyond the known combined effect of age and *HTT* CAG repeat count, models including gene expansion carriers only were run with age and CAG repeat count as covariates. To investigate intergroup differences in outcomes of interest, we applied generalised linear regression models estimated via ordinary least squares. Models were fitted with the variable of interest as the dependent variable, and first with age and group membership, and then with age and CAG and group membership as independent variables. We report results for the omnibus group membership main effect test and relevant contrasts (i.e., healthy controls vs. PreHD and PreHD vs. manifest HD). Other comparisons (i.e., healthy controls vs. manifest HD) were not undertaken as we treat HD as a biological continuum. To investigate alterations unique to later stages of the disease we used clinical measures of disease progression (cUHDRS) and/or cumulative mHTT toxicity (DBS). Tests were not adjusted for multiple comparisons, but the statistical approach was designed to minimise multiplicity within each question of independent interest.

We used Pearson's partial correlations adjusted for age, and for age and CAG to study associations between outcomes of interest and DBS, clinical and imaging measures in gene expansion carriers. Pearson's correlations were used to study associations between metabolites in CSF and plasma. Bias‐corrected and accelerated bootstrapped 95% confidence intervals (95% CI) were calculated for correlation coefficients. We followed Cohen (1992) to qualify the strength of the associations. We calculated interclass‐correlation coefficients (ICC) and 95% CI using two‐way mixed‐effects models to investigate within‐subject short‐term stability. Post hoc sample size calculations for two 2‐group comparisons (i.e., healthy control vs. PreHD and PreHD vs. manifest HD) using 2‐sided Student *t* tests were performed to inform future studies using the detected effect sizes adjusted for age in primary and secondary outcomes, and assuming a 5% type 1 error, 20% type 2 error (80% power) and equal‐sized groups. No adjustments were made for multiplicity (i.e., the alpha level was not adjusted to account for multiple comparisons or hypotheses).

### 2.10 Role of funding source

Funders had no role in study design, data collection, analysis, or interpretation, or writing of the report. The corresponding author had full access to data and final responsibility for the decision to submit for publication.

## 3 RESULTS

### 3.1 Demographics

Eighty participants were recruited: all contributed CSF; 2 (3%) manifest HD were missing plasma. Full cohort characteristics are presented in Table S1. Disease groups were well‐matched for gender and differed as expected in clinical, cognitive and imaging measures. Age differed significantly between groups due to the control group (50.68 years ± 11.0) being matched to all gene expansion carriers, and manifest HD (56.02 years ± 9.36) being more advanced in their disease course than preHD (42.38 years ± 11.04), as previously reported (Byrne et al., 2018b; Rodrigues et al., 2020).

### 3.2 Primary outcomes

**FIGURE 2:**
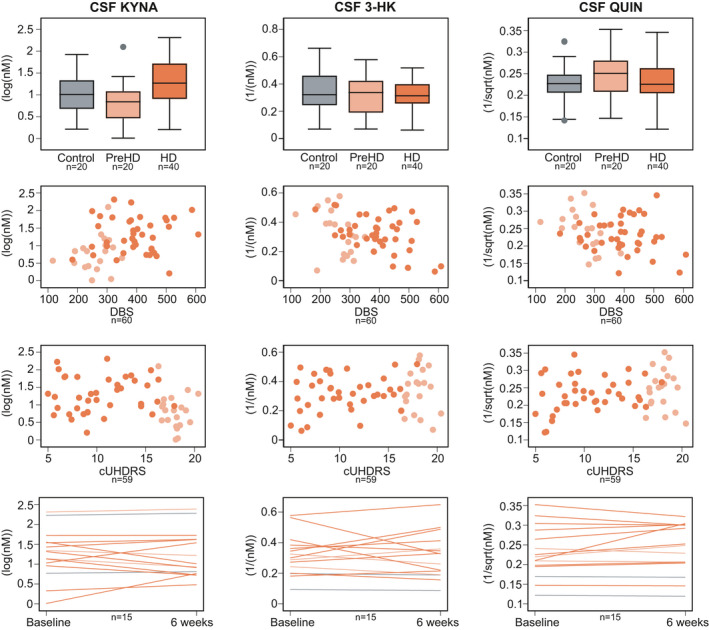
Intergroup differences (top row), associations with Disease Burden Score (DBS, second row) and composite Unified Huntington's Disease Rating Scale (cUHDRS, third row), and within‐subject short‐term stability (bottom row) for primary outcomes: cerebrospinal fluid (CSF) kynurenic acid (KYNA), CSF 3‐hydroxykynurenine (3‐HK) and CSF quinolinic acid (QUIN). Associations were not found in any of the analyses. See Tables 2, S2 and S3 for point estimates and 95% CI. Grey represents healthy controls, light orange preHD and dark orange HD

All three primary outcomes—CSF KYNA, 3‐HK and QUIN—were associated with age in healthy controls, but not with gender, CAG repeat count (gene expansion carriers only), CSF haemoglobin or time in freezer storage (Figure S5). Age‐ and age and CAG‐adjusted CSF levels of KYNA, 3‐HK and QUIN showed no significant difference between groups (Figure 2; Table 2).

**TABLE 2:** Intergroup comparisons

	Healthy controls			Premanifest HD			Manifest HD			Adjusted for	Group membership	HC vs. PM	PM vs. M
	*n*	Mean	*SD*	*n*	Mean	*SD*	*n*	Mean	*SD*		*p* value	*p* value	*p* value
Primary outcomes													
CSF KYNA *log(nM)*	20	0.98	0.46	20	0.80	0.50	40	1.29	0.50	Age	0.154	0.910	0.113
										Age and CAG	*N*/A	*N*/A	0.253
CSF 3‐HK *1/nM*	20	0.35	0.16	20	0.33	0.15	40	0.32	0.12	Age	0.383	0.199	0.231
										Age and CAG	*N*/A	*N*/A	**0.034**
CSF QUIN *1/√nM*	20	0.23	0.05	20	0.25	0.06	40	0.23	0.05	Age	0.312	0.905	0.206
										Age and CAG	*N*/A	*N*/A	0.156
Secondary outcomes													
CSF 3‐HK:KYNA *1/√ratio*	20	0.95	0.27	20	0.85	0.24	40	1.05	0.25	Age	0.094	0.371	**0.036**
										Age and CAG	*N*/A	*N*/A	**0.007**
CSF KYNA:KYN *log(ratio)*	20	−2.79	0.36	20	−2.87	0.33	40	−2.49	0.33	Age	0.005	0.830	**0.007**
										Age and CAG	*N*/A	*N*/A	**0.027**
CSF 3‐HK:KYN *1/(ratio^2^)*	20	238.82	143.04	20	186.91	109.88	40	209.78	113.86	Age	0.445	0.223	0.629
										Age and CAG	*N*/A	*N*/A	0.109
Exploratory outcomes													
CSF TRP μ*M*	20	2.16	0.42	20	2.0	0.45	40	2.05	0.35	Age	0.265	0.980	0.196
										Age and CAG	*N*/A	*N*/A	0.391
CSF KYN *1/√nM*	20	0.15	0.03	20	0.16	0.03	40	0.15	0.02	Age	0.836	0.932	0.682
										Age and CAG	*N*/A	*N*/A	0.699
CSF AA *log(nM)*	20	0.92	0.29	20	0.87	0.37	40	0.94	0.27	Age	0.676	0.682	0.386
										Age and CAG	*N*/A	*N*/A	0.641
Plasma TRP *√*μ*M*	20	7.11	0.81	20	7.18	0.77	38	7.00	0.51	Age	0.642	0.730	0.368
										Age and CAG	*N*/A	*N*/A	0.517
Plasma KYN *log(*μ*M)*	20	0.62	0.26	20	0.66	0.24	38	0.60	0.24	Age	0.408	0.453	0.183
										Age and CAG	*N*/A	*N*/A	0.058
Plasma KYNA *log(nM)*	19	4.07	0.33	19	4.13	0.42	32	3.96	0.32	Age	0.201	0.516	0.086
										Age and CAG	*N*/A	*N*/A	**0.032**
Plasma 3‐HK *log(nM)*	19	3.67	0.24	20	3.58	0.29	38	3.58	0.29	Age	0.442	0.493	0.664
										Age and CAG	*N*/A	*N*/A	0.304
Plasma AA *1/√nM*	18	0.36	0.05	16	0.36	0.05	37	0.34	0.06	Age	0.451	0.500	0.710
										Age and CAG	*N*/A	*N*/A	0.440
Plasma QUIN *log(nM)*	20	5.85	0.36	19	5.80	0.42	38	5.74	0.32	Age	0.148	0.722	0.087
										Age and CAG	*N*/A	*N*/A	0.061
Post hoc exploratory outcome													
CSF KYN/TRP *√ratio*	20	4.57	0.59	20	4.43	0.64	40	4.71	0.68	Age	0.968	0.895	0.931
										Age and CAG	*N*/A	*N*/A	0.752

Note

Note that for outcomes where a transformation includes a reciprocal (i.e., 1/x), an increase in the raw measurement corresponds to a decrease in the transformed value and vice‐versa. Bold values are *p* values smaller than 0.05.

3‐HK, 3‐hydroxykynurenine; AA, anthranilic acid; CSF, cerebrospinal fluid; HC, healthy controls; KYN, kynurenine; KYNA, kynurenic acid; M, manifest HD; *N*/A, not applicable; PM, premanifest HD; QUIN, quinolinic acid; *SD*, standard deviation; TRP, tryptophan.

**FIGURE 3:**
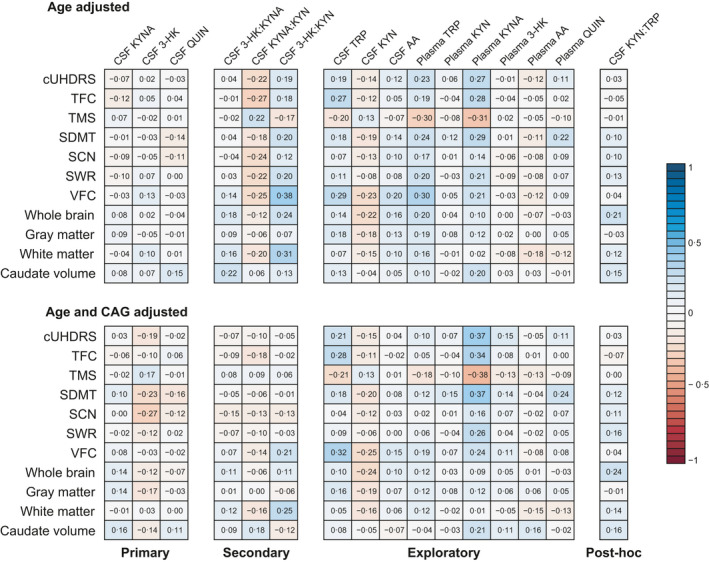
Associations between outcomes and clinical and imaging measures for primary, secondary, exploratory and post hoc outcomes in gene expansion carriers. Numbers and colors are Pearson's partial correlations coefficients adjusted for age (top matrix), and for age and CAG (bottom matrix; see Table S2 for 95% CI and Figure S9 for scatter plots). 3‐HK, 3‐hydroxykynurenine; AA, anthranilic acid; CSF, cerebrospinal fluid; cUHDRS, composite Unified Huntington's Disease Rating Scale; KYN, kynurenine; KYNA, kynurenic acid; QUIN, quinolinic acid; SCN, Stroop Color Naming; SDMT, Symbol Digit Modalities Test; SWR, Stroop Word Reading; TFC, UHDRS Total Functional Capacity; TMS, UHDRS Total Motor Score; TRP, tryptophan; VFC, verbal fluency—Categorical

Associations between age‐adjusted CSF levels of KYNA, 3‐HK or QUIN and DBS, clinical and imaging measures were negligible to weak (Figure 3, Table S2 and Figure S9).

Within‐subject short‐term stability was good for CSF KYNA (ICC 0.84, 95% CI 0.58 to 0.94) and QUIN (ICC 0.90, 95% CI 0.72 to 0.96), and moderate for 3‐HK (ICC 0.68, 95% CI 0.27 to 0.88, Figure 2 and Table S3).

Power calculations showed that the number of participants per arm needed to give 80% power to detect a difference at the alpha level of 0.05 in age‐adjusted CSF levels between healthy controls and preHD would be 48,921 for KYNA, 376 for 3‐HK and 43,665 for QUIN. For the comparison between preHD and HD the number would be 368 for KYNA, 648 for 3‐HK and 579 for QUIN.

### 3.3 Secondary outcomes

**FIGURE 4:**
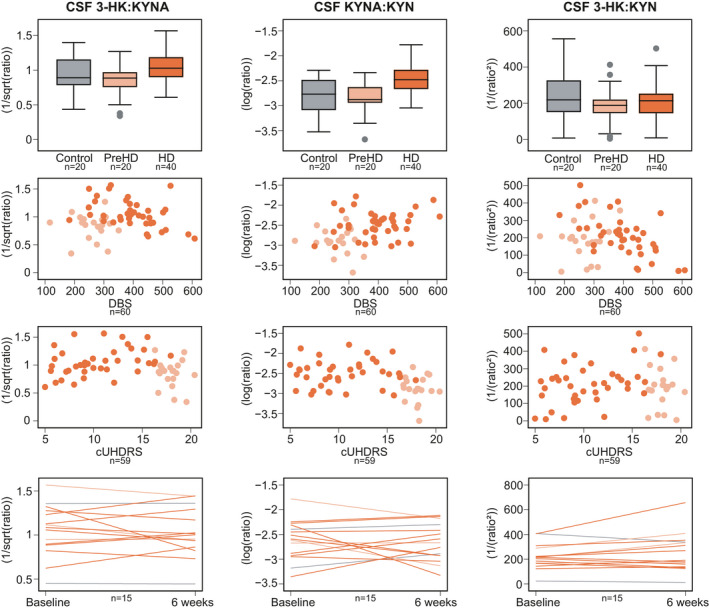
Intergroup differences (top row), associations with Disease Burden Score (DBS, second row) and composite Unified Huntington's Disease Rating Scale (cUHDRS, third row), and within‐subject short‐term stability (bottom row) for secondary outcomes: ratios of cerebrospinal fluid (CSF) 3‐hydroxykynurenine to kynurenic acid (3‐HK:KYNA), CSF kynurenic acid to kynurenine (KYNA:KYN) and CSF 3‐hydroxykynurenine to kynurenine (3‐HK:KYN). Note that for outcomes where a transformation includes a reciprocal (i.e., 1/x), an increase in the raw measurement corresponds to a decreased in the transformed value and vice‐versa. See Tables 2, S2 and S3 for point estimates and 95% CI. Grey represents healthy controls, light orange preHD and dark orange HD

None of the three predefined ratios of interest—CSF 3‐HK:KYNA, KYNA:KYN and 3‐HK:KYN—were associated with age, gender, CAG repeat count, CSF haemoglobin or time in the freezer (Figure S6). Age‐ and age and CAG‐adjusted CSF levels of 3‐HK:KYNA, KYNA:KYN and 3‐HK:KYN showed no difference between healthy controls and preHD (Figure 4; Table 2). There were differences in ratios of 3‐HK:KYNA and KYNA:KYN between preHD and manifest HD (age‐adjusted *p* values = 0.036 and 0.007, respectively; age‐ and CAG‐adjusted *p* values = 0.007 and 0.027, respectively). No differences were found for 3‐HK:KYN.

Associations between age‐adjusted CSF ratios and DBS, clinical and imaging measures were negligible to weak for 3‐HK:KYNA, weak for KYNA:KYN, and weak to moderate for 3‐HK:KYN (Figure 3, Table S2 and Figure S9).

Within‐subject short‐term stability was good for 3‐HK:KYNA (ICC 0.78, 95% CI 0.46 to 0.92) and 3‐HK:KYN (ICC 0.78, 95% CI 0.47 to 0.92), and weak to moderate for KYNA:KYN (ICC 0.43, 95% CI −0.09 to 0.76, Figure 4 and Table S3).

Power calculations showed that the number of participants per arm needed to give 80% power to detect a difference at the alpha level of 0.05 in age‐adjusted CSF levels between healthy controls and preHD would be 777 for 3‐HK:KYNA, 13,521 for KYNA:KYN and 417 for 3‐HK:KYN. For the comparison between preHD and HD the number would be 207 for 3‐HK:KYNA, 126 for KYNA:KYN and 3,993 for 3‐HK:KYN.

### 3.4 Exploratory and post hoc outcomes

Associations between age, gender, CAG repeat count, CSF haemoglobin or time in the freezer are shown in Figures S7 and S10. Age‐ and age and CAG‐adjusted levels of CSF and plasma pre‐defined exploratory measures (individual metabolites) and post hoc analysis (KYN:TRP ratio) showed no difference between groups (Figures S8 and S10 and Table 2). While plasma KYN, 3‐HK, AA and QUIN showed negligible to weak associations with DBS, clinical and imaging measures; CSF AA and CSF KYN:TRP showed negligible to moderate associations, and CSF TRP, KYN, and plasma TRP and KYNA showed weak to moderate associations (Figures S8–S11 and Table S2). Within‐subject short‐term stability varied from low (plasma KYNA) to high (plasma QUIN) and is summarised in Table S3.

### 3.5 Association between CSF and plasma

**FIGURE 5:**
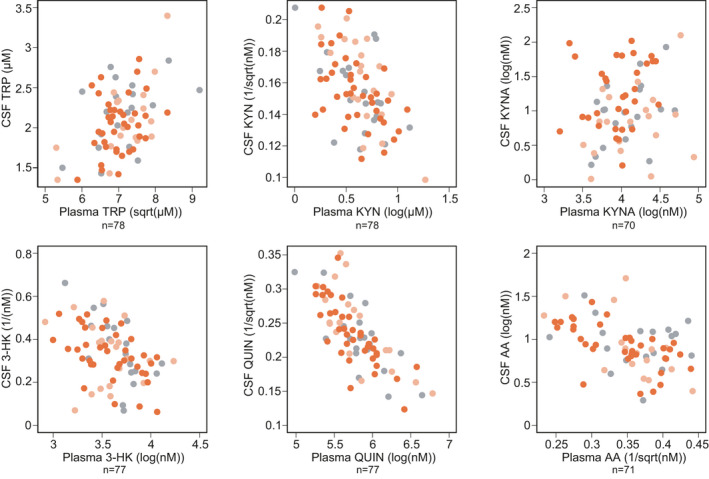
Associations between CSF and plasma. See Table S4 for point estimates and 95% CI. Grey represents healthy controls, light orange preHD and dark orange HD

The matched CSF and plasma analytes were associated, with the exception of KYNA (Figure 5 and Table S4). The strongest association was seen for QUIN (*r* = −0.75, 95% CI −0.83 to −0.65).

## 4 DISCUSSION

In this comprehensive study of KP metabolites in CSF and plasma in HD, we found that TRP, KYN, KYNA, 3‐HK, AA and QUIN were readily and reliably quantifiable in both biofluids in controls and gene expansion carriers. However, we found little evidence to support a substantial derangement of the KP in HD, at least to the extent that it is reflected by the levels of the metabolites in patient‐derived biofluids.

Reports on HD (Rodrigues et al., 2018) and other neurodegenerative conditions such as Alzheimer's and Parkinson's disease (Sorgdrager et al., 2019) have shown CSF KP derangements. Nonetheless, none of the three CSF metabolites we prespecified as being of particular interest—KYNA, 3‐HK and QUIN—had a significantly altered level in preHD or HD. We found no association between CSF levels of these metabolites and any robust clinical, cognitive and MR brain volumetric measures. These findings suggest that the characteristics of KP metabolites make them less likely to be useful as diagnostic and monitoring biomarkers, especially when compared with high performing analytes such as CSF mHTT, and CSF and plasma neurofilament light chain (Byrne et al., 2017a; Byrne et al., 2018b; Rodrigues et al., 2020).

We did find significant associations between all three and age, which might (along with lesser assay reliability and sample quality) be one reason why previous studies reported some alterations, albeit inconsistently. Careful examination and adjustment for confounding variables is especially important given that HD is characterised by an extended premanifest period followed by a long period of manifest disease. This tends to make premanifest cohorts younger than those with manifest HD, and makes it very challenging to recruit a control group that matches the HD groups well for age. We previously showed that an apparent increase in CSF TREM2 in HD was in fact an artefact of its relationship with age (Byrne et al., 2018a).

We predefined three CSF metabolite ratios that might change in the presence of specific predicted derangements of the KP—3‐HK:KYNA as a reflection of the ratio of the main protective and neurotoxic metabolites whose synthetic enzymes have been implicated in HD; KYNA:KYN as a measure of the activity of the KATs and 3‐HK:KYN as a measure of the activity of KMO. We found no evidence for altered CSF 3‐HK:KYN ratio. While there was no evidence of CSF 3‐HK:KYNA and KYNA:KYN ratio differences between healthy controls and preHD, HD seems to have a lower 3‐HK:KYNA ratio than preHD (shown as a higher 1/√ratio) and higher KYNA:KYN (shown as a higher log(ratio)), differences that remained significant after adjustment for age and CAG. Note that for outcomes where a transformation includes a reciprocal (i.e., 1/x), an increase in the raw measurement corresponds to a decreased in the transformed value and vice‐versa. This suggests that the activity of KATs may be increased in HD, an increase that appears to grow as the disease progresses. This may reflect a primary effect of the HD mutation on KATs, or compensatory overactivity because of alterations in the central KP. In a post hoc exploratory analysis of KYN:TRP ratio, which reflects the activity of IDO1/2 and TDO enzymes, we did not see group differences or associations with other disease markers.

As expected, the CSF and plasma levels of each metabolite were significantly associated, and the plasma levels of each were higher, in line with previous reports in Alzheimer's disease and depression (Haroon et al., 2020; Jacobs et al., 2019). There was a positive association between CSF and plasma concentrations for all analytes. Unexpectedly, given previous reports of poor blood‐brain barrier permeability (Fukui et al., 1991), QUIN showed the stronger association between the biofluids. Although further exploitation is needed, this may be explained by disease‐related phenomena implicated in barrier function, differential compartment production and elimination, and parallel derangements in the CNS and periphery. KYNA showed weak associations, while TRP, KYN, 3‐HK, AA were moderate.

Apart from plasma KYN, all measured metabolites were relatively stable over a short period of time of 4 to 8 weeks. Although against the hypothesis of intermittent pathway activation, only a study with higher sampling frequency could answer this question.

Though largely negative, our findings do not imply that the KP in general, or KMO in particular, is not a valid therapeutic target. It is possible that cell‐specific or region‐specific disease‐related alterations in this pathway contribute substantially to the pathogenesis of HD and that pharmacologically correcting them could favourably modify the course of the disease. In that context, it is therefore still possible that measuring KP metabolite levels in CSF could provide one or more valuable readouts of target engagement or meaningful biological effect, for instance by increasing the level of protective substances to above the baseline or control level. The nonsignificant, small differences between groups could also prove robust if tested in a much larger sample set; we have offered sample size calculations for such an experiment. Overall, however, this study provides little support for a relevant alteration of KP function in HD that is biochemically detectable in accessible patient biofluids.

## CONFLICT OF INTEREST

FBR, LMB, AJL, RT, EBJ, RIS, EJW are University College London employees. MA is a University College London Hospitals NHS Foundation Thrust employee. EDV is a King's College London employee. MH and GF are full‐time employees of Charles River Laboratories. FG is an employee of the University of Leicester. FG has the following patent pending ‘KYNURENINE 3‐MONOOXYGENASE (KMO) INHIBITORS, AND USES AND COMPOSITIONS THEREOF’. FBR has provided consultancy services to GLG and F. Hoffmann‐La Roche Ltd. LMR has provided consultancy services to GLG, F. Hoffmann‐La Roche Ltd, Genentech and Annexon. RIS has undertaken consultancy services for Ixico Ltd. EJW reports grants from Medical Research Council (MRC), CHDI Foundation, and F. Hoffmann‐La Roche Ltd during the conduct of the study; personal fees from Hoffman La Roche Ltd, Triplet Therapeutics, PTC Therapeutics, Shire Therapeutics, Wave Life Sciences, Mitoconix, Takeda, Loqus23. All honoraria for these consultancies were paid through the offices of UCL Consultants Ltd., a wholly owned subsidiary of University College London. University College London Hospitals NHS Foundation Trust has received funds as compensation for conducting clinical trials for Ionis Pharmaceuticals, Pfizer and Teva Pharmaceuticals.

## AUTHOR CONTRIBUTIONS

EJW designed the study with the input of FG. FBR and LMB were involved in participant recruitment. Eligibility, clinical examinations and sample collection were performed by FBR, LMB, and RT. Imaging assessments were conceived RIS, EBJ and EDV, data were acquired by EDV, MA, EBJ, and processed by RIS and EBJ. MM and GF processed and analysed the patient samples. FBR developed and performed the statistical analysis; FBR, AJL and EJW interpreted the data and wrote the manuscript; and all authors contributed to reviewing the manuscript.

## Supporting information

Supplementary Material

## Data Availability

The data that support the findings of this study are available on request from the corresponding author, EJW. The data are not publicly available due to their containing information that could compromise the privacy of research participants.
