# Supplementary material for: Kynurenine pathway metabolites in cerebrospinal fluid and blood as potential biomarkers in Huntington's disease
Source: J Neurochem. 2021 May 5;158(2):539–53. doi: 10.1111/jnc.15360 (PMC8375100; doi:10.1111/jnc.15360)
Supplement: Supplementary file 1 — Supplementary Material [file JNC-158-539-s001.pdf]

Kynurenine pathway metabolites in cerebrospinal fluid and blood as potential biomarkers in Huntington's disease

Filipe B Rodrigues[1†], Lauren M Byrne[1†], Alexander J Lowe[1], Rosanna Tortelli[1], Mariette Heins[2], Gunnar Flik[2], Eileanoir B Johnson[1], Enrico De Vita[3,4], Rachael I Scahill[1], Flaviano Giorgini[5] and Edward J Wild[1*]

1 UCL Huntington's Disease Centre, UCL Queen Square Institute of Neurology, University College London, London, UK

2 Charles River Laboratories, Groningen, the Netherlands

3 Lysholm Department of Neuroradiology, National Hospital for Neurology and Neurosurgery, London, UK

4 Department of Biomedical Engineering, School of Biomedical Engineering and Imaging Sciences, King's College London, UK

5 Department of Genetics and Genome Biology, University of Leicester, Leicester, UK

[†] These authors contributed equally as first authors of this study

***** Dr Edward J Wild, e.wild@ucl.ac.uk, UCL Huntington's Disease Centre, University College London, 10-12 Russell Square, London WC1B 5EH

Running title: Kynurenine pathway in Huntington's disease

Supplementary material

Supplementary tables

Table S1 Cohort characteristics. CAG, CAG repeat count; DBS, Disease Burden Score; cUHDRS, composite Unified Huntington’s Disease Rating Scale; N/A, not applicable; SD, standard deviation; SDMT, Symbol Digit Modalities Test; SCN, Stroop Color Naming; SWR, Stroop Word Reading; TFC, UHDRS Total Functional Capacity; TMS, UHDRS Total Motor Score; VFC, Verbal Fluency – Categorical.

	**All Participants**			**Healthy Controls**			**Premanifest HD**			**Manifest HD**		
	**n**	**Mean**	**SD**	**n**	**Mean**	**SD**	**n**	**Mean**	**SD**	**n**	**Mean**	**SD**
Demographic												
Gender	80	F38/M42		20	F10/M10		20	F10/M10		40	F18/M22	
Age (years)	80	51.27	11.55	20	50.68	11.03	20	42.38	11.04	40	56.02	9.36
Clinical scores												
CAG	60	42.50	2.03	N/A	N/A	N/A	20	42.00	1.62	40	42.75	2.18
DBS	60	352.80	104.30	N/A	N/A	N/A	20	267.20	61.92	40	395.60	94.64
cUHDRS	79	14.12	4.509	20	17.40	1.49	20	17.98	1.10	39	10.46	3.57
TFC	80	11.20	2.62	20	13.00	0.00	20	13.00	0.00	40	9.40	2.70
TMS	80	20.05	22.34	20	2.35	2.43	20	2.80	2.80	40	37.52	19.43
Cognitive tests												
SDMT	79	40.39	17.28	20	50.90	10.38	20	55.55	9.32	39	27.23	12.60
VFC	80	19.04	6.84	20	24.30	4.12	20	23.30	3.42	40	14.28	5.83
SCN	80	62.10	22.01	20	75.80	13.07	20	81.30	10.12	40	45.65	16.90
SWR	80	81.08	29.22	20	100.20	17.36	20	105.00	11.75	40	59.55	23.63
Imaging volumes adjusted for total intracranial volume (mL)												
Whole brain	64	1121.19	156.02	15	1173.93	115.97	16	1233.56	156.20	33	1042.73	128.99
Caudate volume	63	5.26	1.80	15	6.75	1.38	16	6.29	1.21	32	4.05	1.35
Grey matter	64	648.72	79.49	15	705.93	51.61	16	709.01	46.38	33	593.49	61.39
White matter	64	407.61	41.61	15	439.59	32.64	16	430.41	28.14	33	382.01	34.00

Table S2 Associations between outcomes and disease burden score (DBS), clinical and imaging measures for (A) primary, (B) secondary , (C) exploratory and (D) post-hoc outcomes in gene expansion carriers. 3-HK, 3-hydroxykynurenine; AA, Anthranilic acid; CSF, cerebrospinal fluid; cUHDRS, composite Unified Huntington's Disease Rating Scale; KYN, kynurenine; KYNA, kynurenic acid; r, Pearson's partial correlation coefficient; QUIN, quinolinic acid; SCN, Stroop Color Naming; SDMT, Symbol Digit Modalities Test; SWR, Stroop Word Reading; TFC, UHDRS Total Functional Capacity; TMS, UHDRS Total Motor Score; TRP, tryptophan; VFC, Verbal Fluency – Categorical.

A

		Age-adjusted					Age and CAG-adjusted				
		n	r	95% confidence interval		p-value	n	r	95% confidence interval		p-value
**CSF KYNA**											
	DBS	60	0.14	-0.12	0.38	0.265	60	0.01	-0.26	0.34	0.961
	cUHDRS	59	-0.07	-0.34	0.19	0.616	59	0.03	-0.24	0.27	0.829
	TFC	60	-0.12	-0.36	0.13	0.337	60	-0.06	-0.31	0.20	0.666
	TMS	60	0.07	-0.18	0.31	0.564	60	-0.02	-0.25	0.22	0.862
	SDMT	59	-0.01	-0.27	0.24	0.967	59	0.10	-0.15	0.33	0.432
	VFC	60	-0.03	-0.26	0.20	0.833	60	0.08	-0.15	0.30	0.509
	SCN	60	-0.09	-0.36	0.19	0.530	60	0.00	-0.28	0.24	0.988
	SWR	60	-0.10	-0.37	0.14	0.430	60	-0.02	-0.30	0.22	0.900
	Whole brain	49	0.08	-0.17	0.31	0.533	49	0.14	-0.06	0.35	0.191
	Caudate volume	48	0.08	-0.16	0.31	0.503	48	0.16	-0.06	0.38	0.150
	Grey matter	49	0.09	-0.14	0.32	0.447	49	0.14	-0.08	0.36	0.205
	White matter	49	-0.04	-0.34	0.23	0.794	49	-0.01	-0.30	0.23	0.955
**CSF 3-HK**											
	DBS	60	-0.28	-0.51	0.01	0.033	60	-0.11	-0.45	0.16	0.476
	cUHDRS	59	0.02	-0.23	0.28	0.857	59	-0.19	-0.40	0.04	0.093
	TFC	60	0.05	-0.18	0.31	0.664	60	-0.10	-0.36	0.13	0.418
	TMS	60	-0.02	-0.24	0.21	0.851	60	0.17	-0.04	0.37	0.105
	SDMT	59	-0.03	-0.27	0.24	0.834	59	-0.23	-0.42	-0.01	0.031
	VFC	60	0.13	-0.12	0.36	0.284	60	-0.03	-0.27	0.20	0.814
	SCN	60	-0.05	-0.31	0.21	0.692	60	-0.27	-0.45	-0.05	0.009
	SWR	60	0.07	-0.18	0.35	0.607	60	-0.12	-0.35	0.11	0.301
	Whole brain	49	0.02	-0.27	0.29	0.910	49	-0.12	-0.36	0.10	0.295
	Caudate volume	48	0.07	-0.20	0.35	0.618	48	-0.14	-0.34	0.11	0.238
	Grey matter	49	-0.05	-0.31	0.22	0.728	49	-0.17	-0.40	0.03	0.114
	White matter	49	0.10	-0.16	0.35	0.441	49	0.03	-0.21	0.25	0.779
**CSF QUIN**											
	DBS	60	-0.02	-0.29	0.24	0.858	60	-0.16	-0.43	0.08	0.206
	cUHDRS	59	-0.03	-0.31	0.25	0.832	59	-0.02	-0.32	0.23	0.865
	TFC	60	0.04	-0.27	0.32	0.801	60	0.06	-0.27	0.33	0.690
	TMS	60	0.01	-0.23	0.24	0.949	60	-0.01	-0.24	0.20	0.947
	SDMT	59	-0.14	-0.41	0.12	0.299	59	-0.16	-0.40	0.09	0.202
	VFC	60	-0.03	-0.28	0.24	0.822	60	-0.02	-0.27	0.22	0.881
	SCN	60	-0.11	-0.38	0.15	0.425	60	-0.12	-0.37	0.12	0.344
	SWR	60	0.00	-0.30	0.27	0.992	60	0.02	-0.28	0.29	0.889
	Whole brain	49	-0.04	-0.36	0.22	0.773	49	-0.07	-0.37	0.18	0.595
	Caudate volume	48	0.15	-0.12	0.38	0.245	48	0.11	-0.14	0.32	0.341
	Grey matter	49	-0.01	-0.30	0.26	0.965	49	-0.03	-0.33	0.23	0.829
	White matter	49	0.01	-0.29	0.27	0.938	49	0.00	-0.31	0.26	0.974

B

	Age-adjusted					Age and CAG-adjusted				
	n	r	95% confidence interval		p-value	n	r	95% confidence interval		p-value
**CSF 3-HK:KYNA**										
DBS	60	-0.19	-0.45	0.11	0.190	60	-0.20	-0.42	0.05	0.100
cUHDRS	59	0.04	-0.25	0.28	0.766	59	-0.07	-0.29	0.13	0.519
TFC	60	-0.01	-0.24	0.25	0.957	60	-0.09	-0.32	0.14	0.416
TMS	60	-0.02	-0.24	0.28	0.891	60	0.08	-0.14	0.29	0.444
SDMT	59	0.04	-0.19	0.26	0.714	59	-0.05	-0.25	0.18	0.621
VFC	60	0.14	-0.13	0.38	0.270	60	0.07	-0.17	0.28	0.518
SCN	60	-0.04	-0.31	0.19	0.770	60	-0.15	-0.38	0.06	0.176
SWR	60	0.03	-0.26	0.26	0.790	60	-0.07	-0.32	0.11	0.531
Whole brain	49	0.18	-0.07	0.42	0.136	49	0.11	-0.11	0.30	0.321
Caudate volume	48	0.22	0.01	0.42	0.036	48	0.09	-0.11	0.26	0.369
Grey matter	49	0.09	-0.12	0.32	0.431	49	0.01	-0.20	0.19	0.884
White matter	49	0.16	-0.15	0.42	0.266	49	0.12	-0.21	0.37	0.423
**CSF KYNA:KYN**										
DBS	60	0.24	0.01	0.46	0.039	60	0.06	-0.25	0.31	0.692
cUHDRS	59	-0.22	-0.45	0.04	0.079	59	-0.10	-0.34	0.15	0.425
TFC	60	-0.27	-0.47	-0.03	0.016	60	-0.18	-0.39	0.05	0.107
TMS	60	0.22	-0.05	0.43	0.077	60	0.09	-0.12	0.33	0.407
SDMT	59	-0.18	-0.41	0.06	0.127	59	-0.06	-0.30	0.19	0.654
VFC	60	-0.25	-0.46	0.01	0.038	60	-0.14	-0.36	0.11	0.243
SCN	60	-0.24	-0.48	0.04	0.066	60	-0.13	-0.41	0.14	0.379
SWR	60	-0.22	-0.47	0.03	0.093	60	-0.10	-0.38	0.14	0.472
Whole brain	49	-0.12	-0.39	0.15	0.382	49	-0.06	-0.27	0.20	0.637
Caudate volume	48	0.06	-0.18	0.27	0.584	48	0.18	-0.11	0.40	0.173
Grey matter	49	-0.06	-0.29	0.20	0.628	49	0.00	-0.20	0.17	0.992
White matter	49	-0.20	-0.47	0.08	0.158	49	-0.16	-0.42	0.11	0.234
**CSF 3-HK:KYN**										
DBS	60	-0.41	-0.61	-0.16	<0.001	60	-0.23	-0.45	0.02	0.058
cUHDRS	59	0.19	-0.08	0.42	0.139	59	-0.05	-0.30	0.22	0.728
TFC	60	0.18	-0.07	0.39	0.127	60	-0.02	-0.27	0.20	0.897
TMS	60	-0.17	-0.39	0.13	0.180	60	0.06	-0.19	0.31	0.637
SDMT	59	0.20	-0.05	0.41	0.093	59	-0.01	-0.26	0.30	0.931
VFC	60	0.38	0.13	0.57	0.001	60	0.21	-0.03	0.43	0.072
SCN	60	0.12	-0.15	0.34	0.330	60	-0.13	-0.38	0.09	0.272
SWR	60	0.20	-0.08	0.42	0.115	60	-0.03	-0.27	0.21	0.777
Whole brain	49	0.24	-0.10	0.48	0.094	49	0.11	-0.13	0.37	0.393
Caudate volume	48	0.13	-0.11	0.37	0.289	48	-0.12	-0.31	0.09	0.254
Grey matter	49	0.07	-0.23	0.35	0.629	49	-0.06	-0.26	0.20	0.575
White matter	49	0.31	-0.02	0.55	0.031	49	0.25	-0.05	0.54	0.096

C

		Age-adjusted					Age and CAG-adjusted				
		n	r	95% confidence interval		p-value	n	r	95% confidence interval		p-value
**CSF TRP**											
	DBS	60	-0.10	-0.35	0.14	0.428	60	-0.13	-0.37	0.05	0.203
	cUHDRS	59	0.19	-0.09	0.45	0.167	59	0.21	-0.06	0.43	0.104
	TFC	60	0.27	0.05	0.47	0.010	60	0.28	0.07	0.47	0.006
	TMS	60	-0.20	-0.47	0.08	0.143	60	-0.21	-0.45	0.08	0.106
	SDMT	59	0.18	-0.14	0.45	0.234	59	0.18	-0.11	0.43	0.189
	VFC	60	0.29	0.06	0.46	0.004	60	0.32	0.10	0.47	0.001
	SCN	60	0.07	-0.22	0.29	0.595	60	0.04	-0.23	0.27	0.766
	SWR	60	0.11	-0.15	0.31	0.376	60	0.09	-0.17	0.28	0.439
	Whole brain	49	0.14	-0.21	0.41	0.384	49	0.10	-0.23	0.40	0.520
	Caudate volume	48	0.13	-0.20	0.41	0.409	48	0.08	-0.25	0.38	0.630
	Grey matter	49	0.18	-0.16	0.52	0.295	49	0.16	-0.21	0.48	0.389
	White matter	49	0.07	-0.19	0.30	0.593	49	0.05	-0.21	0.29	0.716
**CSF KYN**											
	DBS	60	0.05	-0.19	0.28	0.669	60	0.02	-0.27	0.25	0.878
	cUHDRS	59	-0.14	-0.37	0.12	0.264	59	-0.15	-0.36	0.09	0.217
	TFC	60	-0.12	-0.37	0.15	0.360	60	-0.11	-0.37	0.17	0.411
	TMS	60	0.13	-0.11	0.35	0.260	60	0.13	-0.11	0.32	0.247
	SDMT	59	-0.19	-0.42	0.09	0.162	59	-0.20	-0.41	0.04	0.098
	VFC	60	-0.23	-0.41	-0.01	0.027	60	-0.25	-0.43	-0.03	0.017
	SCN	60	-0.13	-0.37	0.07	0.270	60	-0.12	-0.34	0.05	0.230
	SWR	60	-0.08	-0.32	0.15	0.530	60	-0.06	-0.29	0.15	0.600
	Whole brain	49	-0.22	-0.48	0.02	0.079	49	-0.24	-0.48	0.00	0.046
	Caudate volume	48	-0.04	-0.28	0.20	0.763	48	-0.05	-0.26	0.18	0.663
	Grey matter	49	-0.18	-0.43	0.07	0.183	49	-0.19	-0.45	0.08	0.158
	White matter	49	-0.15	-0.39	0.09	0.209	49	-0.16	-0.38	0.08	0.174
**CSF AA**											
	DBS	60	-0.17	-0.38	0.09	0.158	60	-0.16	-0.38	0.12	0.211
	cUHDRS	59	0.12	-0.15	0.38	0.395	59	0.04	-0.22	0.31	0.768
	TFC	60	0.05	-0.24	0.35	0.742	60	-0.02	-0.34	0.25	0.896
	TMS	60	-0.07	-0.34	0.18	0.596	60	0.01	-0.24	0.25	0.957
	SDMT	59	0.14	-0.08	0.39	0.238	59	0.08	-0.14	0.32	0.508
	VFC	60	0.20	-0.03	0.42	0.088	60	0.15	-0.06	0.35	0.140
	SCN	60	0.10	-0.15	0.37	0.469	60	0.03	-0.22	0.27	0.846
	SWR	60	0.08	-0.19	0.34	0.543	60	0.00	-0.24	0.28	0.981
	Whole brain	49	0.16	-0.10	0.41	0.221	49	0.10	-0.14	0.35	0.426
	Caudate volume	48	0.05	-0.22	0.29	0.722	48	-0.07	-0.27	0.16	0.531
	Grey matter	49	0.13	-0.13	0.43	0.384	49	0.07	-0.19	0.35	0.598
	White matter	49	0.10	-0.15	0.35	0.448	49	0.06	-0.19	0.29	0.623

		Age-adjusted					Age and CAG-adjusted				
		n	r	95% confidence interval		p-value	n	r	95% confidence interval		p-value
**Plasma TRP**											
	DBS	58	-0.27	-0.45	-0.08	0.003	58	-0.09	-0.29	0.15	0.421
	cUHDRS	57	0.23	-0.04	0.45	0.064	57	0.10	-0.21	0.34	0.461
	TFC	58	0.19	-0.07	0.38	0.102	58	0.05	-0.20	0.27	0.665
	TMS	58	-0.30	-0.54	-0.03	0.022	58	-0.18	-0.45	0.14	0.221
	SDMT	57	0.24	-0.04	0.45	0.053	57	0.12	-0.12	0.34	0.307
	VFC	58	0.30	0.01	0.46	0.005	58	0.19	-0.14	0.36	0.102
	SCN	58	0.17	-0.13	0.39	0.197	58	0.02	-0.31	0.26	0.897
	SWR	58	0.20	-0.08	0.41	0.087	58	0.06	-0.24	0.28	0.642
	Whole brain	47	0.20	-0.06	0.47	0.140	47	0.12	-0.21	0.38	0.419
	Caudate volume	46	0.10	-0.28	0.42	0.580	46	-0.04	-0.46	0.30	0.836
	Grey matter	47	0.19	-0.06	0.44	0.146	47	0.12	-0.20	0.36	0.409
	White matter	47	0.16	-0.07	0.39	0.159	47	0.12	-0.14	0.33	0.305
**Plasma KYN**											
	DBS	58	0.02	-0.26	0.33	0.909	58	0.06	-0.24	0.37	0.722
	cUHDRS	57	0.06	-0.22	0.34	0.700	57	0.07	-0.16	0.32	0.566
	TFC	58	-0.04	-0.33	0.28	0.817	58	-0.04	-0.33	0.26	0.764
	TMS	58	-0.08	-0.36	0.20	0.585	58	-0.10	-0.34	0.13	0.408
	SDMT	57	0.12	-0.13	0.39	0.365	57	0.15	-0.10	0.37	0.207
	VFC	58	0.05	-0.20	0.29	0.670	58	0.07	-0.14	0.26	0.508
	SCN	58	0.01	-0.25	0.23	0.961	58	0.01	-0.21	0.22	0.934
	SWR	58	-0.03	-0.26	0.20	0.776	58	-0.04	-0.22	0.15	0.655
	Whole brain	47	0.04	-0.31	0.36	0.814	47	0.03	-0.25	0.32	0.820
	Caudate volume	46	-0.02	-0.32	0.26	0.913	46	-0.03	-0.31	0.20	0.811
	Grey matter	47	0.08	-0.22	0.43	0.630	47	0.08	-0.19	0.41	0.620
	White matter	47	-0.01	-0.32	0.22	0.921	47	-0.02	-0.30	0.23	0.890
**Plasma KYNA**											
	DBS	51	-0.03	-0.29	0.24	0.830	51	-0.04	-0.31	0.35	0.826
	cUHDRS	50	0.27	0.00	0.53	0.050	50	0.37	0.12	0.54	<0.001
	TFC	51	0.28	-0.01	0.54	0.040	51	0.34	0.03	0.56	0.009
	TMS	51	-0.31	-0.53	-0.01	0.022	51	-0.38	-0.55	-0.13	<0.001
	SDMT	50	0.29	0.02	0.56	0.037	50	0.37	0.18	0.56	<0.001
	VFC	51	0.21	-0.04	0.44	0.096	51	0.24	0.01	0.42	0.019
	SCN	51	0.14	-0.12	0.42	0.306	51	0.16	-0.08	0.36	0.157
	SWR	51	0.21	-0.06	0.45	0.108	51	0.26	0.04	0.45	0.010
	Whole brain	40	0.10	-0.26	0.37	0.522	40	0.09	-0.26	0.36	0.544
	Caudate volume	39	0.20	-0.11	0.46	0.172	39	0.21	-0.04	0.43	0.094
	Grey matter	40	0.12	-0.22	0.44	0.475	40	0.12	-0.22	0.40	0.458
	White matter	40	0.02	-0.27	0.27	0.910	40	0.01	-0.30	0.25	0.947

		Age-adjusted					Age and CAG-adjusted				
		n	r	95% confidence interval		p-value	n	r	95% confidence interval		p-value
**Plasma 3-HK**											
	DBS	58	0.19	-0.07	0.41	0.124	58	0.04	-0.21	0.24	0.753
	cUHDRS	57	-0.01	-0.28	0.28	0.971	57	0.15	-0.09	0.40	0.242
	TFC	58	-0.04	-0.33	0.27	0.799	58	0.08	-0.22	0.37	0.575
	TMS	58	0.02	-0.25	0.28	0.887	58	-0.13	-0.36	0.10	0.272
	SDMT	57	0.01	-0.30	0.30	0.958	57	0.14	-0.12	0.41	0.285
	VFC	58	-0.03	-0.26	0.23	0.830	58	0.11	-0.09	0.31	0.267
	SCN	58	-0.06	-0.31	0.23	0.655	58	0.07	-0.16	0.29	0.565
	SWR	58	-0.09	-0.31	0.18	0.494	58	0.04	-0.16	0.25	0.682
	Whole brain	47	0.00	-0.31	0.29	0.975	47	0.05	-0.21	0.35	0.723
	Caudate volume	46	0.03	-0.30	0.29	0.844	46	0.11	-0.17	0.41	0.459
	Grey matter	47	0.02	-0.27	0.30	0.875	47	0.06	-0.22	0.37	0.662
	White matter	47	-0.08	-0.35	0.21	0.592	47	-0.05	-0.33	0.20	0.684
**Plasma AA**											
	DBS	53	0.11	-0.20	0.36	0.428	53	0.06	-0.18	0.29	0.610
	cUHDRS	52	-0.12	-0.36	0.13	0.337	52	-0.05	-0.27	0.18	0.670
	TFC	53	-0.05	-0.32	0.26	0.750	53	0.01	-0.24	0.32	0.963
	TMS	53	-0.05	-0.35	0.22	0.738	53	-0.13	-0.40	0.10	0.318
	SDMT	52	-0.11	-0.35	0.14	0.389	52	-0.04	-0.28	0.18	0.722
	VFC	53	-0.12	-0.39	0.17	0.404	53	-0.08	-0.32	0.17	0.556
	SCN	53	-0.08	-0.32	0.20	0.553	53	-0.02	-0.26	0.24	0.844
	SWR	53	-0.08	-0.33	0.22	0.577	53	-0.02	-0.26	0.23	0.845
	Whole brain	44	-0.07	-0.34	0.22	0.653	44	0.01	-0.26	0.22	0.957
	Caudate volume	43	0.03	-0.21	0.26	0.830	43	0.16	-0.07	0.37	0.163
	Grey matter	44	0.00	-0.29	0.28	0.993	44	0.06	-0.20	0.29	0.619
	White matter	44	-0.18	-0.45	0.07	0.185	44	-0.15	-0.40	0.09	0.250
**Plama QUIN**											
	DBS	57	-0.02	-0.26	0.28	0.897	57	0.09	-0.13	0.35	0.463
	cUHDRS	56	0.11	-0.20	0.38	0.450	56	0.11	-0.17	0.40	0.454
	TFC	57	0.02	-0.28	0.31	0.873	57	0.00	-0.29	0.27	0.995
	TMS	57	-0.10	-0.33	0.15	0.427	57	-0.09	-0.31	0.15	0.464
	SDMT	56	0.22	-0.05	0.46	0.096	56	0.24	-0.06	0.49	0.087
	VFC	57	0.09	-0.18	0.31	0.470	57	0.08	-0.15	0.32	0.506
	SCN	57	0.09	-0.17	0.40	0.552	57	0.07	-0.20	0.41	0.639
	SWR	57	0.07	-0.21	0.39	0.665	57	0.05	-0.25	0.37	0.766
	Whole brain	47	-0.03	-0.33	0.29	0.865	47	-0.03	-0.29	0.28	0.810
	Caudate volume	46	-0.01	-0.25	0.27	0.963	46	-0.02	-0.23	0.21	0.891
	Grey matter	47	0.05	-0.24	0.37	0.742	47	0.05	-0.23	0.34	0.753
	White matter	47	-0.12	-0.38	0.25	0.441	47	-0.13	-0.39	0.23	0.415

D

		Age-adjusted					Age and CAG-adjusted				
		n	r	95% confidence interval		p-value	n	r	95% confidence interval		p-value
**CSF KYN:TRP**											
	DBS	60	-0.01	-0.25	0.23	0.965	60	0.01	-0.24	0.26	0.936
	cUHDRS	59	0.03	-0.24	0.27	0.805	59	0.03	-0.21	0.26	0.782
	TFC	60	-0.05	-0.35	0.17	0.692	60	-0.07	-0.38	0.18	0.641
	TMS	60	-0.01	-0.23	0.23	0.951	60	0.00	-0.24	0.23	0.983
	SDMT	59	0.10	-0.12	0.32	0.378	59	0.12	-0.10	0.36	0.292
	VFC	60	0.10	-0.12	0.29	0.358	60	0.11	-0.13	0.31	0.303
	SCN	60	0.13	-0.07	0.36	0.240	60	0.16	-0.05	0.37	0.157
	SWR	60	0.04	-0.21	0.28	0.764	60	0.04	-0.19	0.32	0.752
	Whole brain	49	0.21	-0.02	0.42	0.061	49	0.24	-0.02	0.46	0.048
	Caudate volume	48	-0.03	-0.23	0.19	0.755	48	-0.01	-0.21	0.22	0.959
	Grey matter	49	0.12	-0.12	0.35	0.338	49	0.14	-0.14	0.39	0.325
	White matter	49	0.15	-0.11	0.37	0.239	49	0.16	-0.08	0.40	0.194

Table S3 Within-subject short-term stability for primary, secondary, exploratory and post-hoc outcomes. 3-HK, 3-hydroxykynurenine; AA, Anthranilic acid; CSF, cerebrospinal fluid; ICC, KYN, kynurenine; KYNA, kynurenic acid; QUIN, quinolinic acid; TRP, tryptophan.

		ICC	95% confidence interval	
**Primary Outcomes**				
	CSF KYNA	0.84	0.58	0.94
	CSF 3-HK	0.68	0.27	0.88
	CSF QUIN	0.90	0.72	0.96
**Secondary Outcomes**				
	CSF 3-HK:KYNA	0.78	0.46	0.92
	CSF KYNA:KYN	0.43	-0.09	0.76
	CSF 3-HK:KYN	0.78	0.47	0.92
**Exploratory Outcomes**				
	CSF TRP	0.58	0.11	0.83
	CSF KYN	0.67	0.25	0.87
	CSF AA	0.84	0.58	0.94
	Plasma TRP	0.58	0.09	0.84
	Plasma KYN	0.54	0.03	0.82
	Plasma KYNA	-0.16	-0.68	0.45
	Plasma 3-HK	0.57	0.08	0.84
	Plasma AA	0.80	0.41	0.94
	Plasma QUIN	0.89	0.69	0.96
**Post-hoc Exploratory Outcome**				
	CSF KYN:TRP	0.57	0.10	0.83

Table S4 Associations and ratios between CSF and plasma. 3-HK, 3-hydroxykynurenine; AA, Anthranilic acid, KYN, kynurenine; KYNA, kynurenic acid; QUIN, quinolinic acid; r, Pearson's partial correlation coefficient; TRP, tryptophan.

		n	r	95% confidence interval		p-value	CSF to plasma ratio
	TRP	78	0.43	0.21	0.60	<0.001	0.04 μM/μM
	KYN	78	-0.55	-0.70	-0.36	<0.001	23.43 nM/μM = 0.02343 nM/nM
	KYNA	70	0.17	-0.13	0.43	0.229	0.06 nM/nM
	3-HK	77	-0.42	-0.57	-0.22	<0.001	0.11 nM/nM
	AA	71	-0.48	-0.63	-0.29	<0.001	0.31 nM/nM
	QUIN	77	-0.75	-0.83	-0.66	<0.001	0.06 nM/nM

Supplementary figures

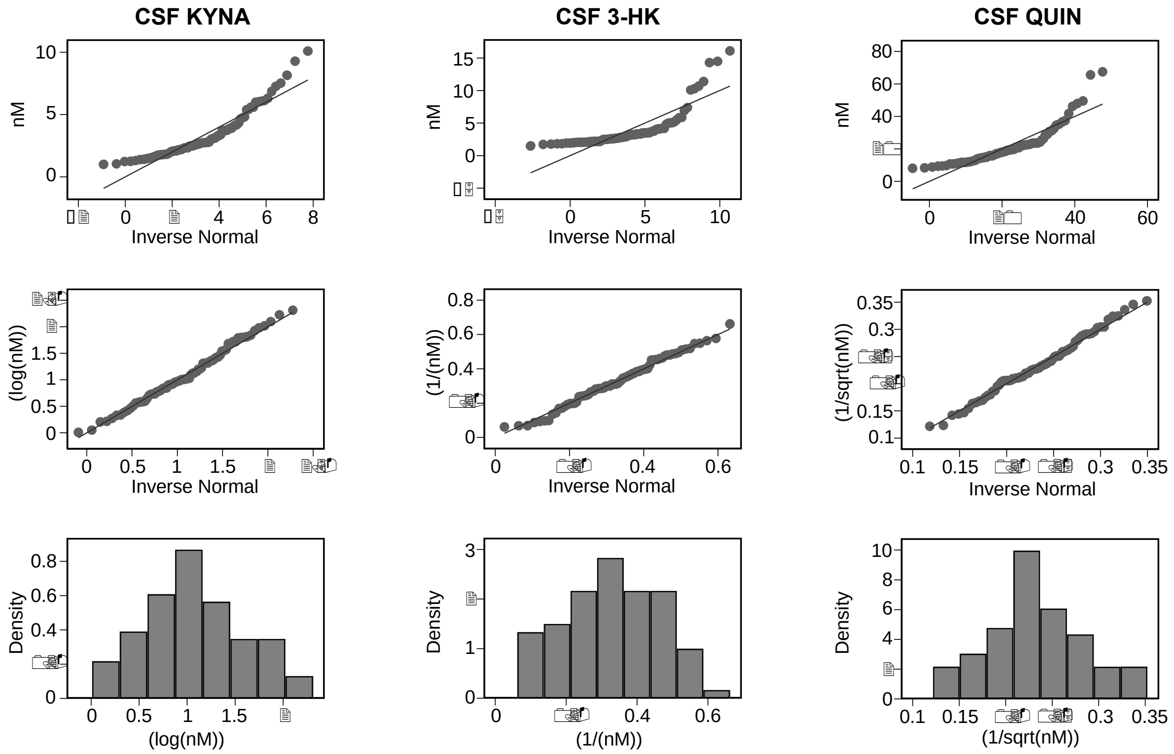

Figure S1 Distributions and arithmetic operations used to transform primary outcomes: cerebrospinal fluid (CSF) kynurenic acid (KYNA), CSF 3-hydroxykynurenine (3-HK) and CSF quinolinic acid (QUIN). Top row depicts a quantile-quantile plot for observed distribution against normal distribution, middle row shows a quantile-quantile plot for transformed distribution against normal distribution, and the bottom row a histogram for transformed distribution.

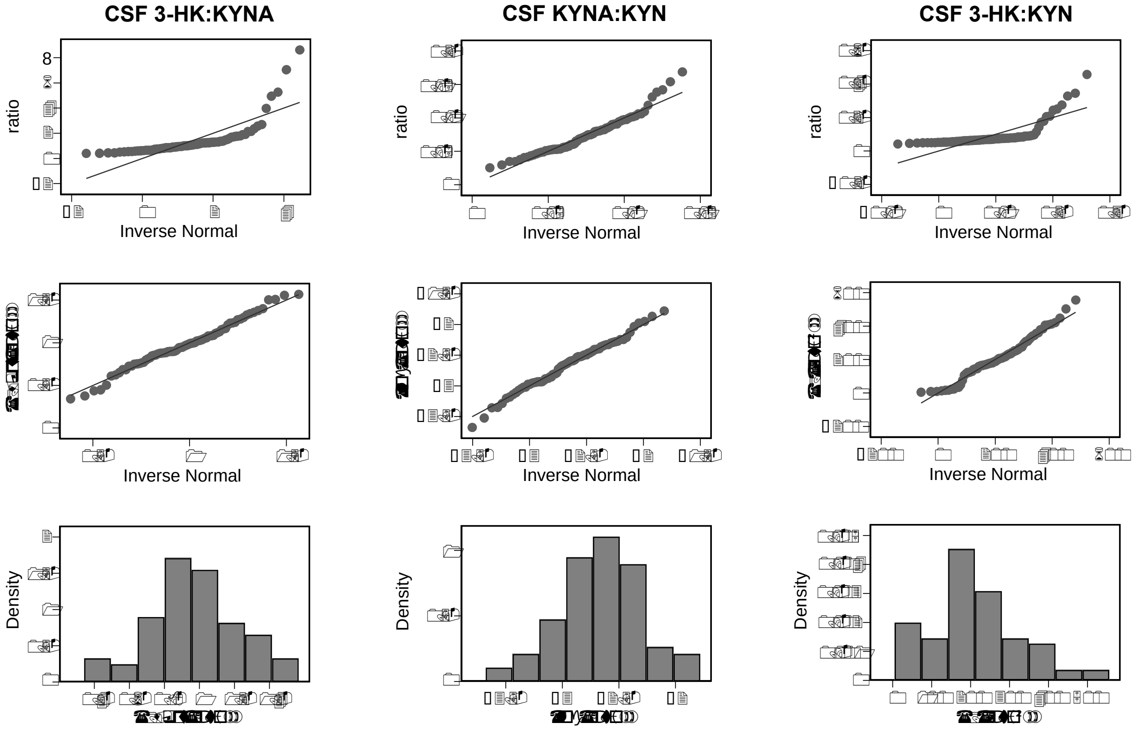

Figure S2 Distributions and arithmetic operations used to transform secondary outcomes: ratios of cerebrospinal fluid (CSF) 3-hydroxykynurenine to kynurenic acid (3-HK:KYNA), CSF kynurenic acid to kynurenine (KYNA:KYN) and CSF 3-hydroxykynurenine to kynurenine (3-HK:KYN). Top row depicts a quantile-quantile plot for observed distribution against normal distribution, middle row shows a quantile-quantile plot for transformed distribution against normal distribution, and the bottom row a histogram for transformed distribution.

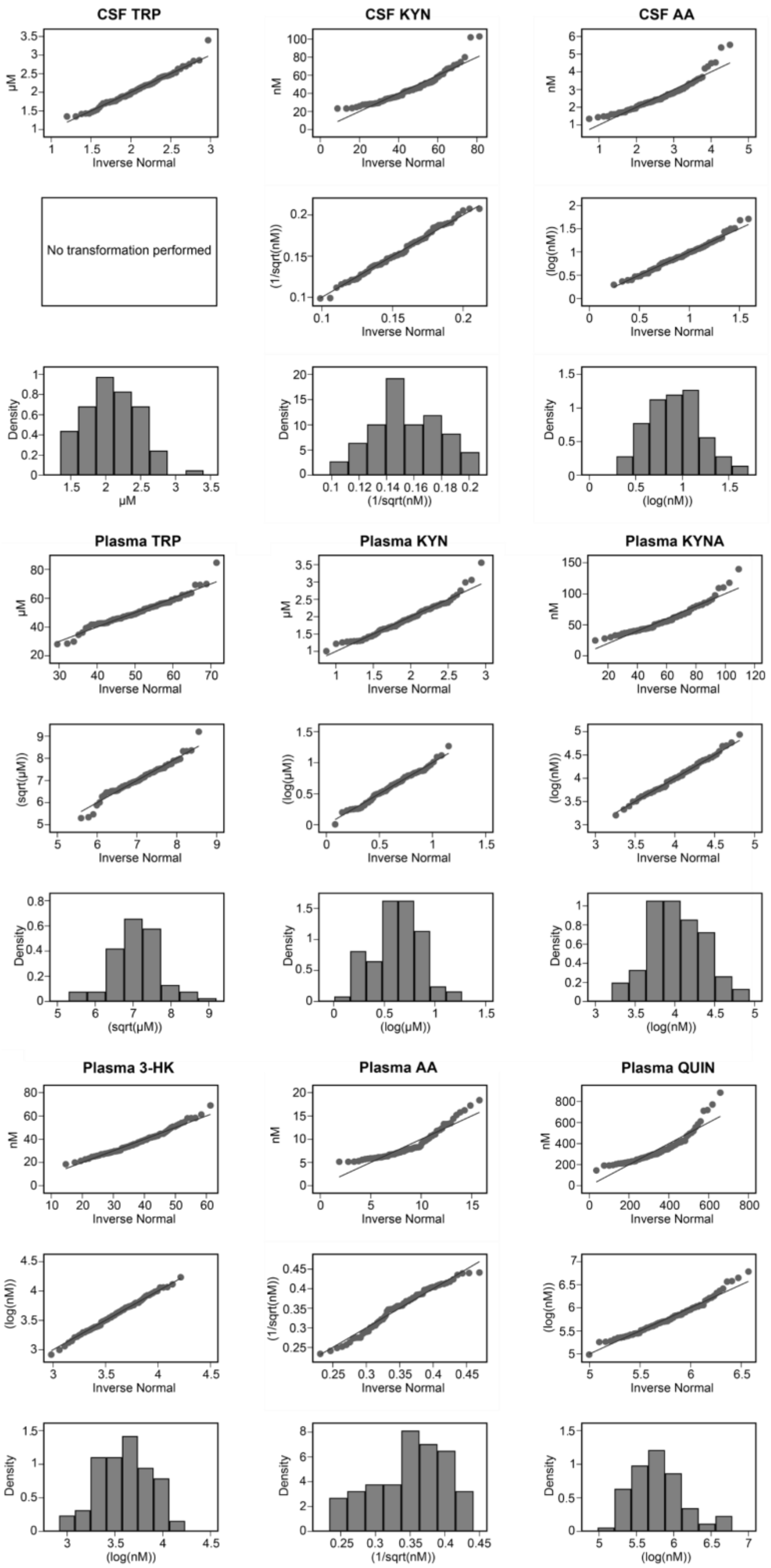

Figure S3 Distributions and arithmetic operations used to transform exploratory outcomes: cerebrospinal fluid (CSF) tryptophan (TRP), CSF kynurenine (KYN), CSF anthranilic acid (AA), plasma TRP, plasma KYN, plasma KYNA, plasma 3-hydroxykynurenine (3-HK), plasma AA, and plasma quinolinic acid (QUIN). In each of the three 3 by 3 subpanels, top row depicts a quantile-quantile plot for observed distribution against normal distribution, middle row shows a quantile-quantile plot for transformed distribution against normal distribution, and the bottom row a histogram for transformed distribution.

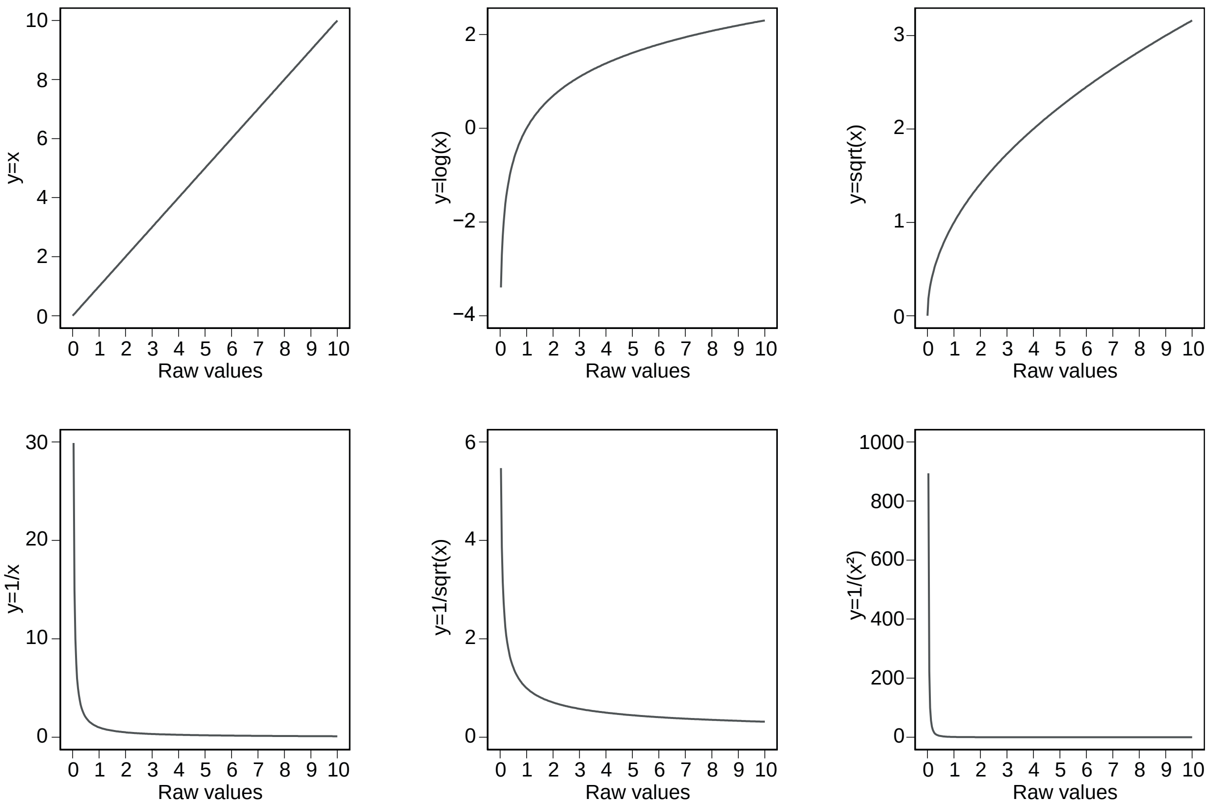

Figure S4 Arithmetic transformations applied to outcomes of interest. Note that for outcomes where a transformation includes a reciprocal (i.e. 1/x), an increase in the raw measurement corresponds to a decrease in the transformed value and vice-versa

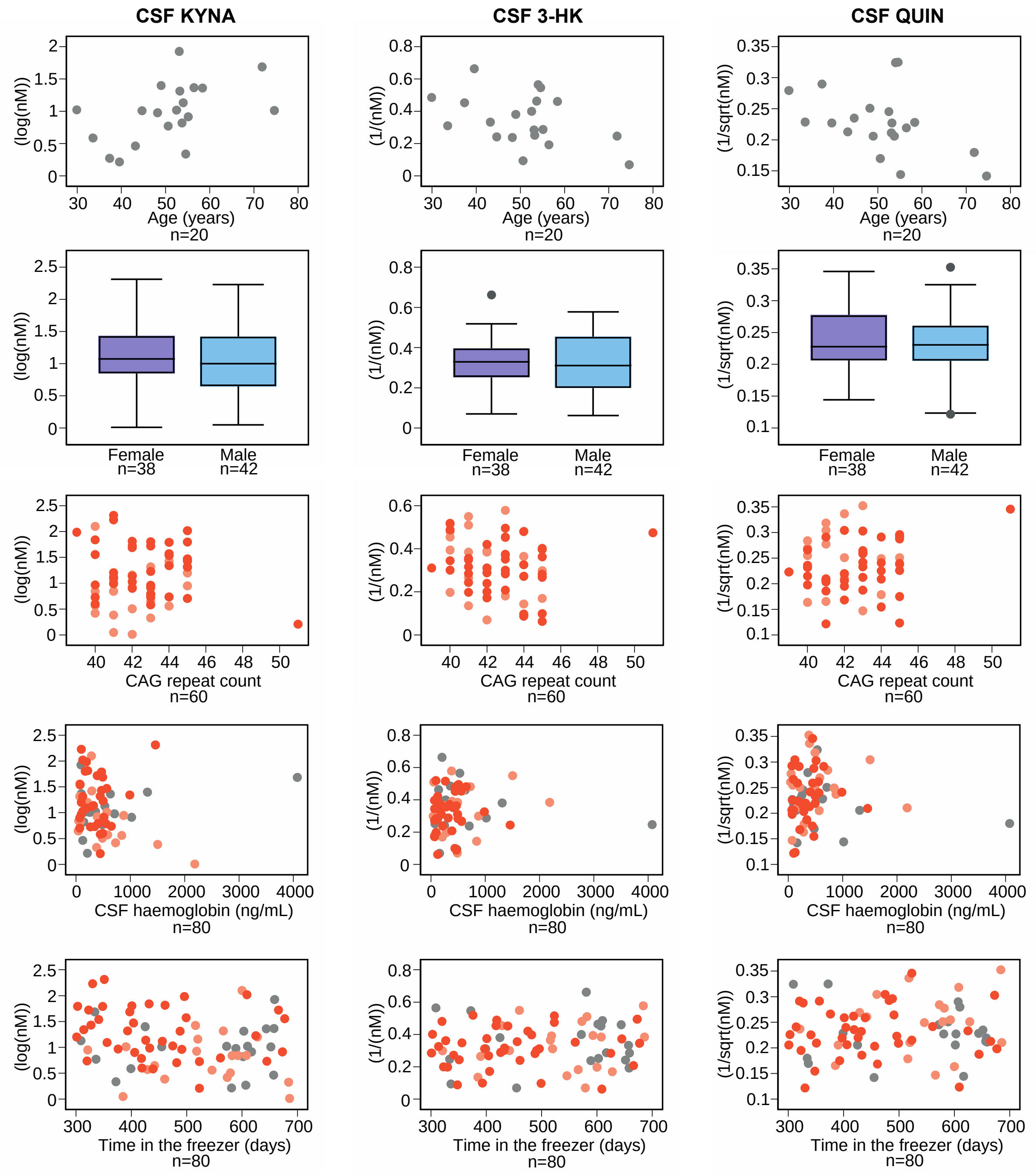

Figure S5 Associations between age (healthy controls only, top row), gender (second row), CAG repeat count (gene expansion carriers only, middle row), CSF haemoglobin (third row) and time in the freezer (bottom row) and primary outcomes: cerebrospinal fluid (CSF) kynurenic acid (KYNA), CSF 3-hydroxykynurenine (3-HK) and CSF quinolinic acid (QUIN). Grey represents healthy controls, light orange preHD and dark orange HD.

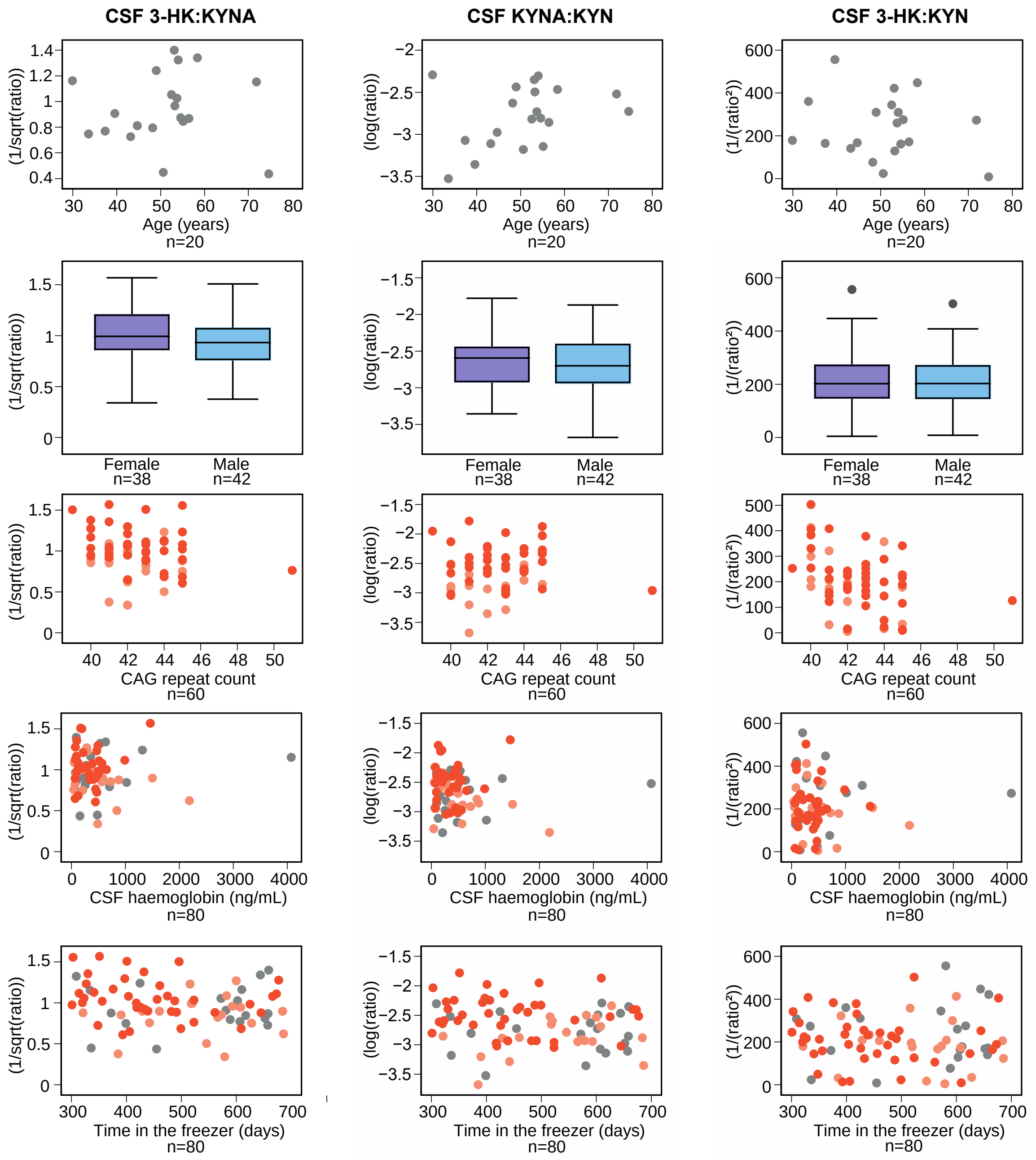

Figure S6 Associations between age (healthy controls only, top row), gender (second row), CAG repeat count (gene expansion carriers only, middle row), CSF haemoglobin (third row) and time in the freezer (bottom row) and secondary outcomes: ratios of cerebrospinal fluid (CSF) 3-hydroxykynurenine to kynurenic acid (3-HK:KYNA), CSF kynurenic acid to kynurenine (KYNA:KYN) and CSF 3-hydroxykynurenine to kynurenine (3-HK:KYN). Grey represents healthy controls, light orange preHD and dark orange HD.

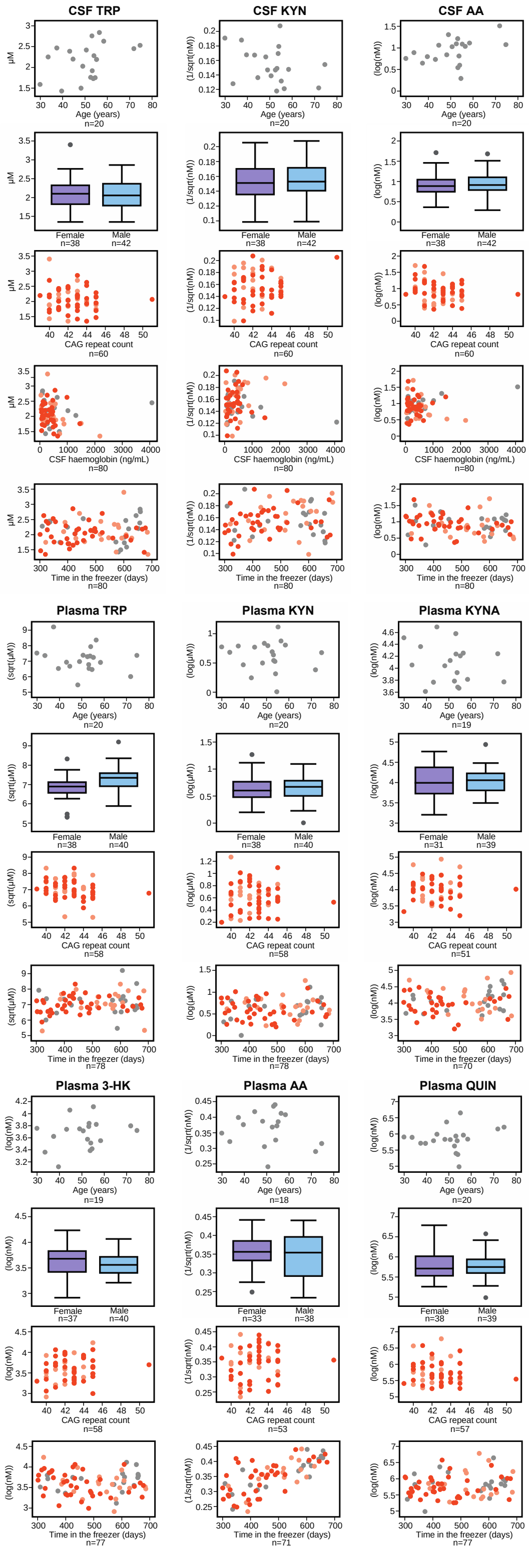

Figure S7 Associations between age (healthy controls only, top row), gender (second row), CAG repeat count (gene expansion carriers only, middle row), CSF haemoglobin (third row) and time in the freezer (bottom row) and exploratory outcomes: cerebrospinal fluid (CSF) tryptophan (TRP), CSF kynurenine (KYN), CSF anthranilic acid (AA), plasma TRP, plasma KYN, plasma KYNA, plasma 3-hydroxykynurenine (3-HK), plasma AA, and plasma quinolinic acid (QUIN). Grey represents healthy controls, light orange preHD and dark orange HD.

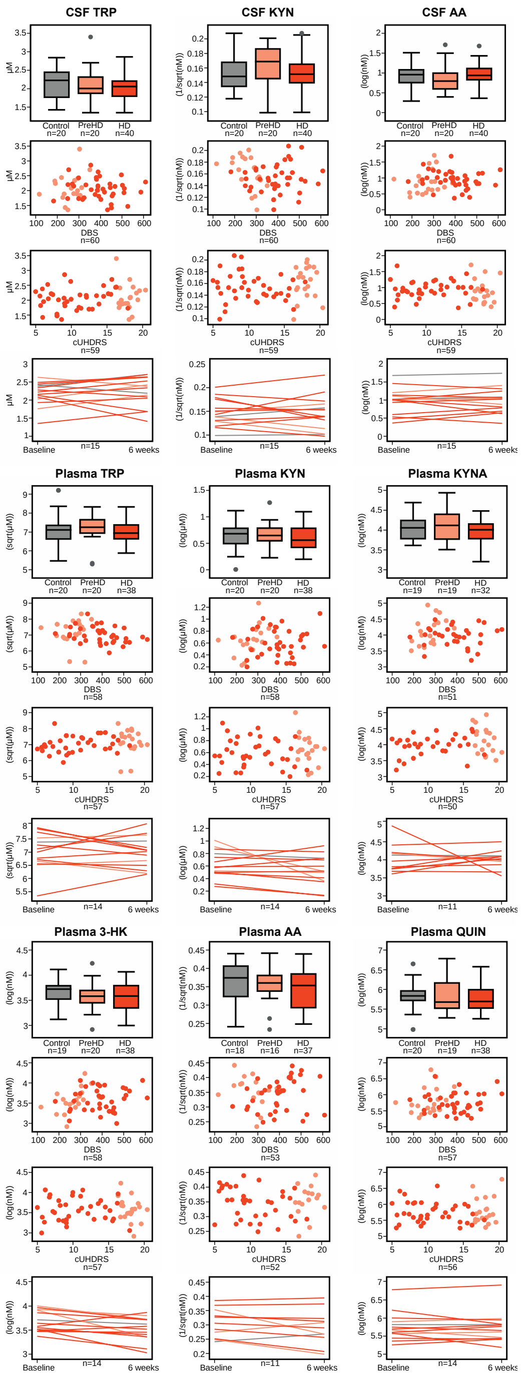

Figure S8 Intergroup differences (top row), associations with Disease Burden Score (DBS, second row) and composite Unified Huntington's Disease Rating Scale (cUHDRS, third row), and within-subject short-term stability (bottom row) for exploratory outcomes: cerebrospinal fluid (CSF) tryptophan (TRP), CSF kynurenine (KYN), CSF anthranilic acid (AA), plasma TRP, plasma KYN, plasma KYNA, plasma 3-hydroxykynurenine (3-HK), plasma AA, and plasma quinolinic acid (QUIN). Grey represents healthy controls, light orange preHD and dark orange HD.

CSF KYNA

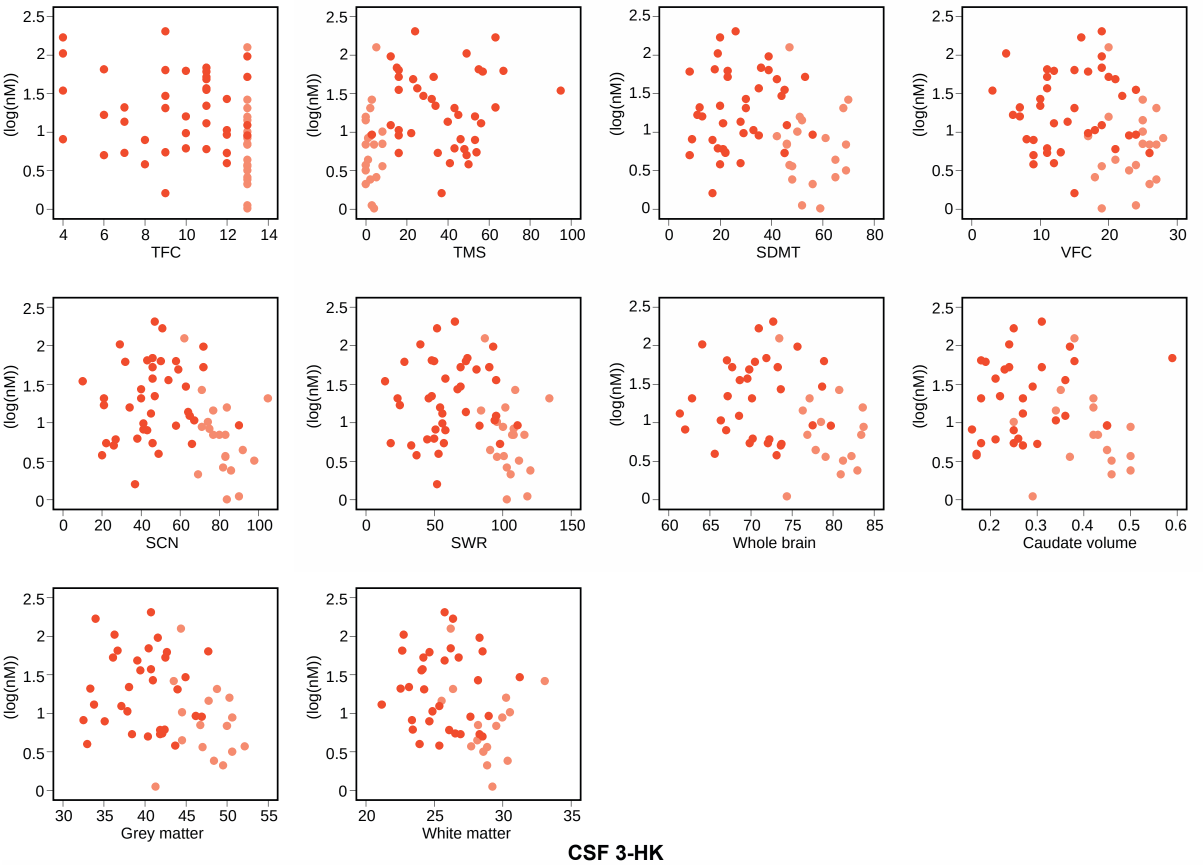

CSF 3-HK

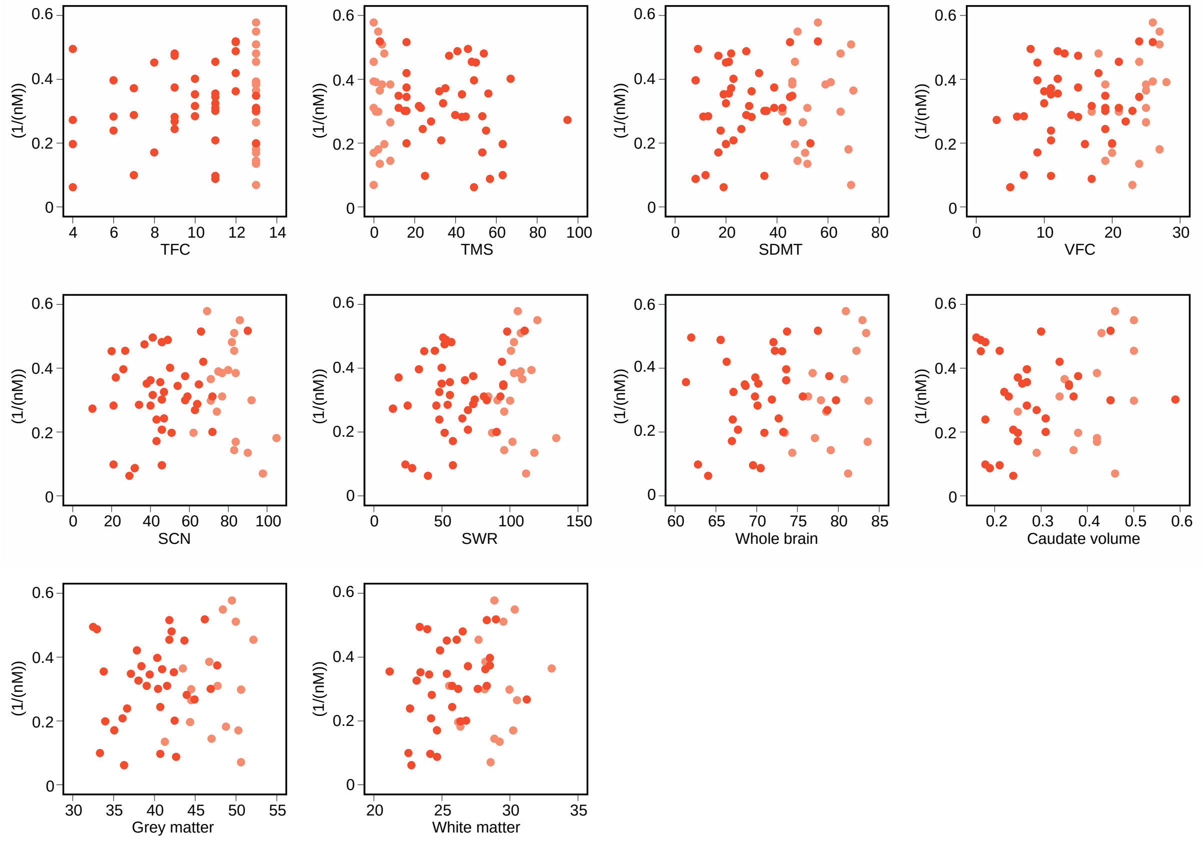

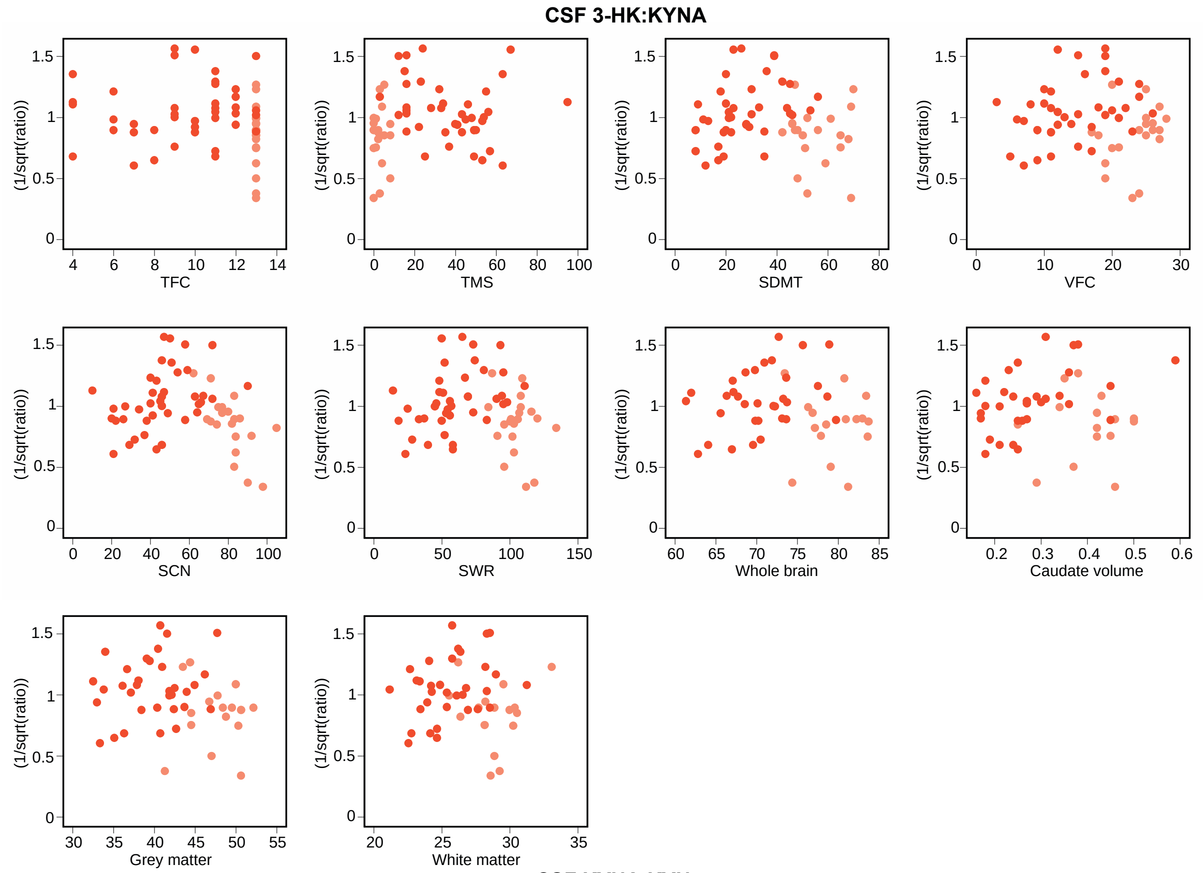
CSF 3-HK:KYNA
(1/sqrt(ratio))
TFC
TMS
SDMT
VFC
SCN
SWR
Whole brain
Caudate volume
Grey matter
White matter

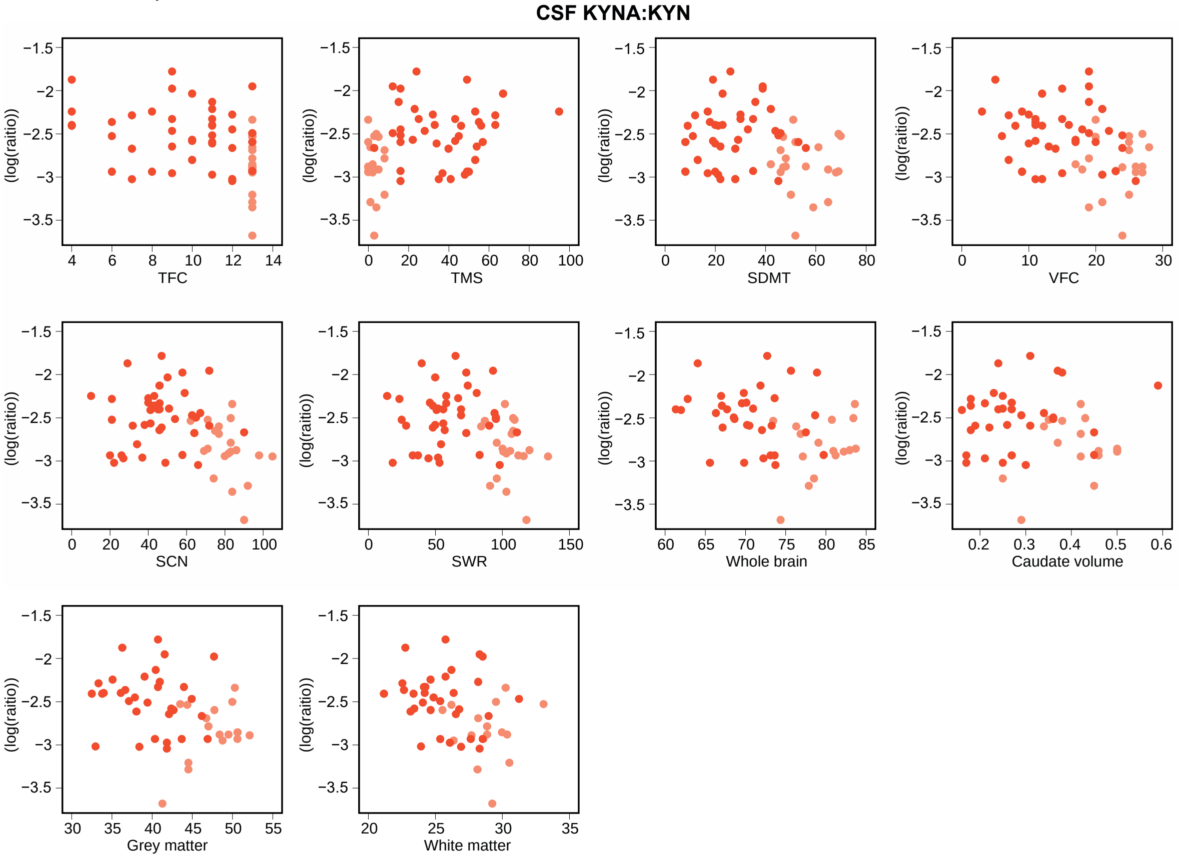
CSF KYNA:KYN
(log(raitio))
TFC
TMS
SDMT
VFC
SCN
SWR
Whole brain
Caudate volume
Grey matter
White matter

CSF 3-HK:KYN

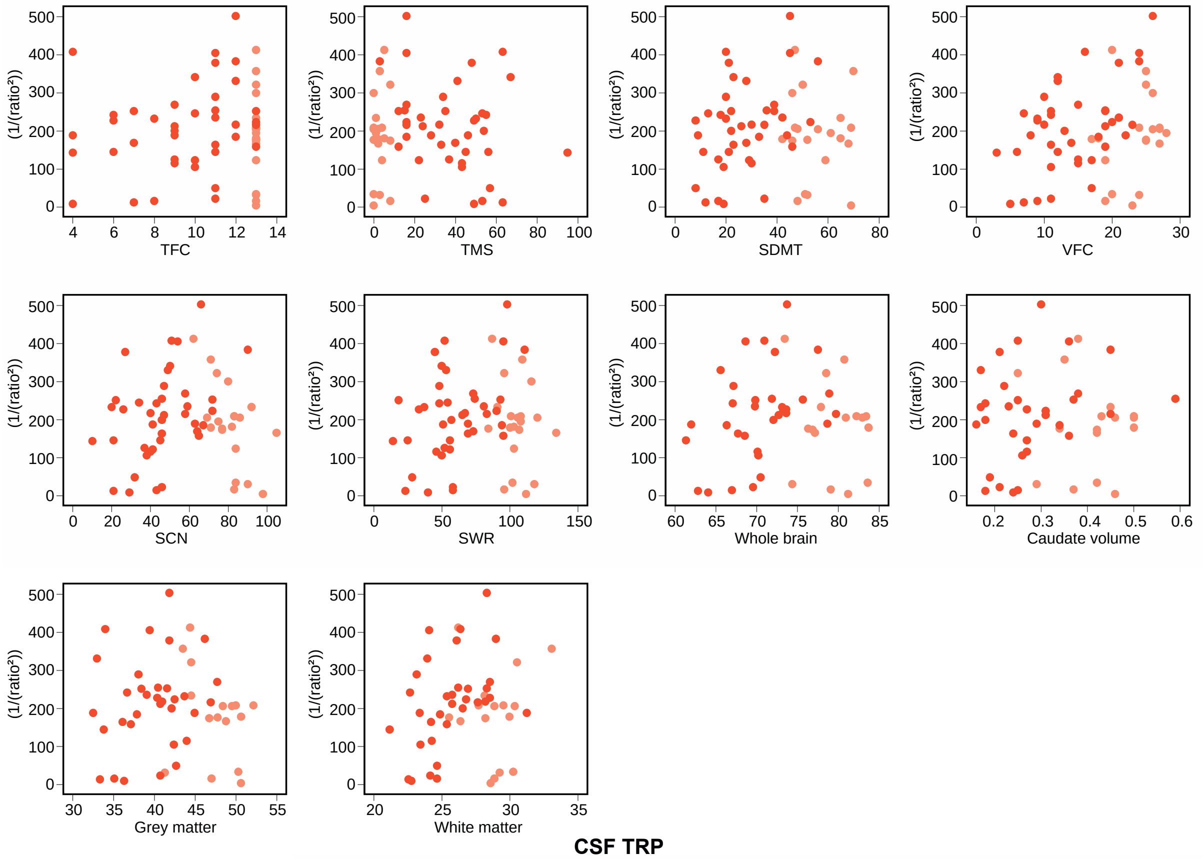

CSF TRP

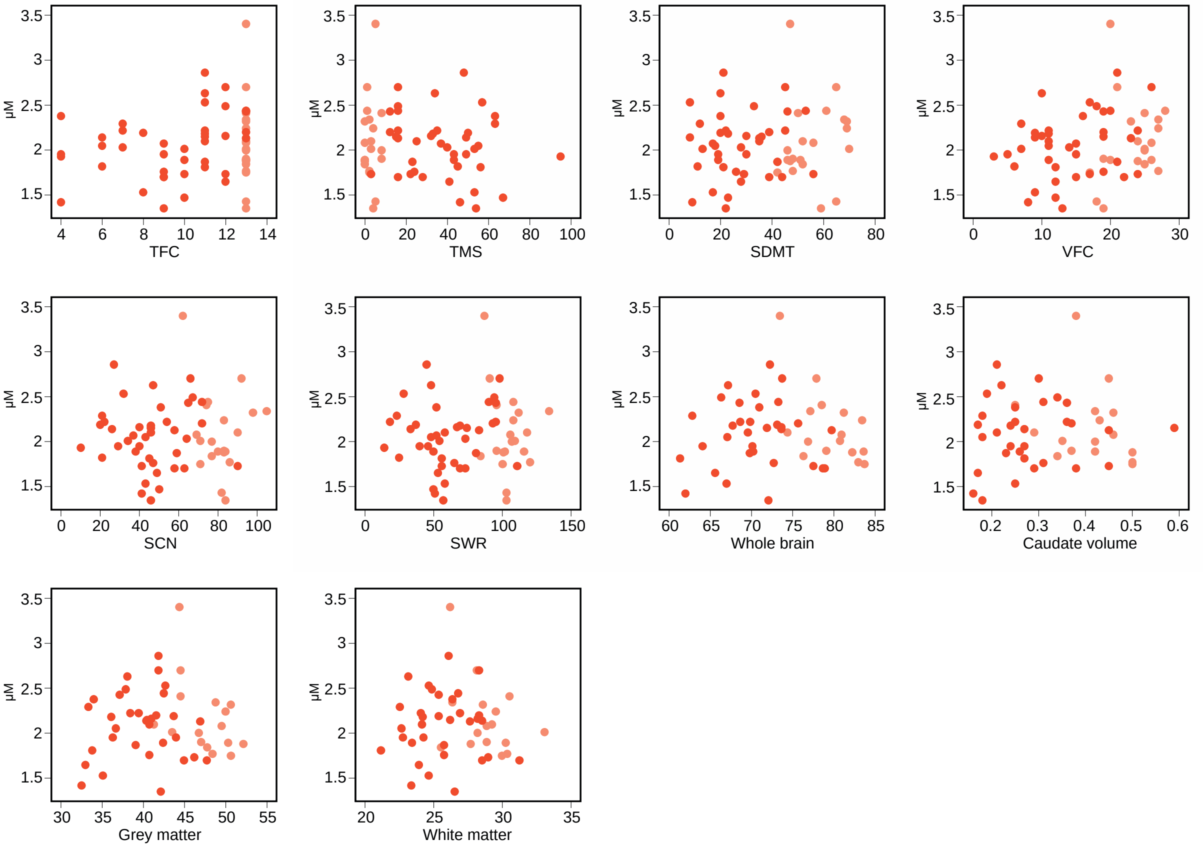

CSF KYN

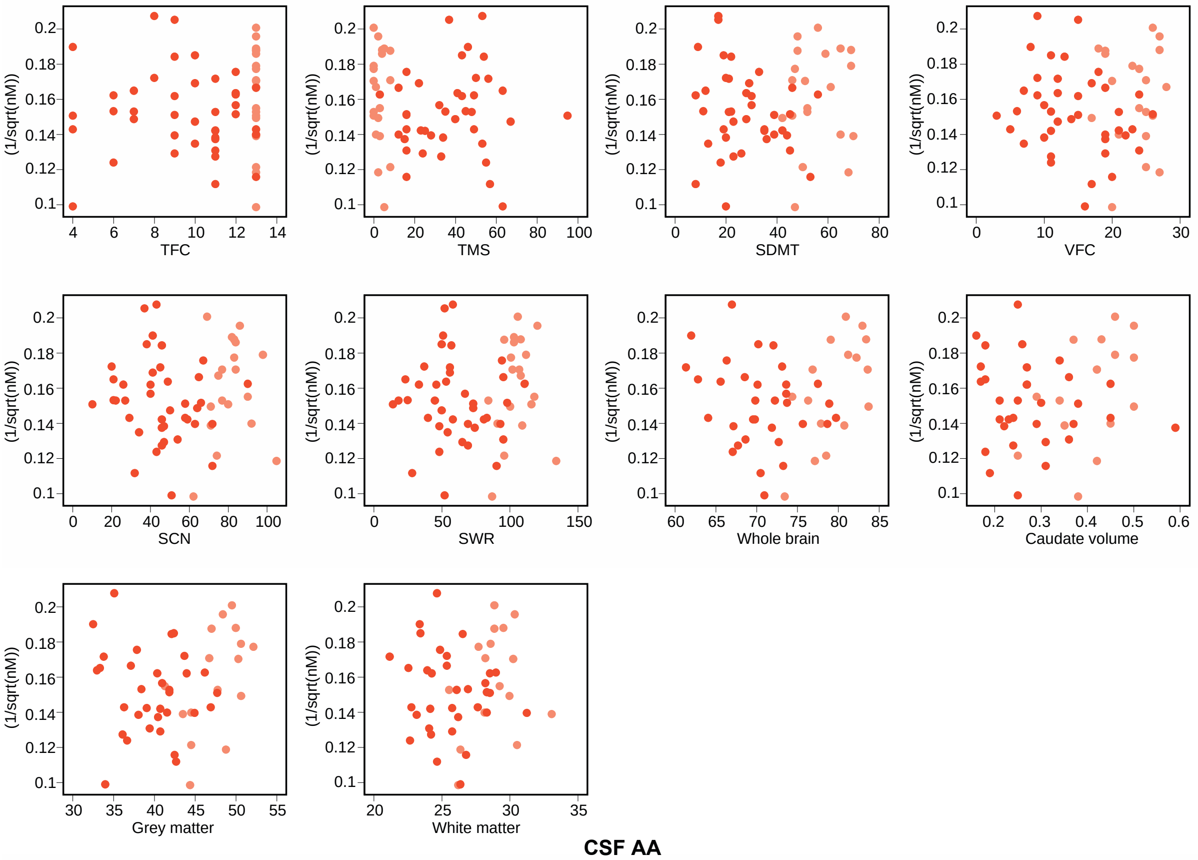

CSF AA

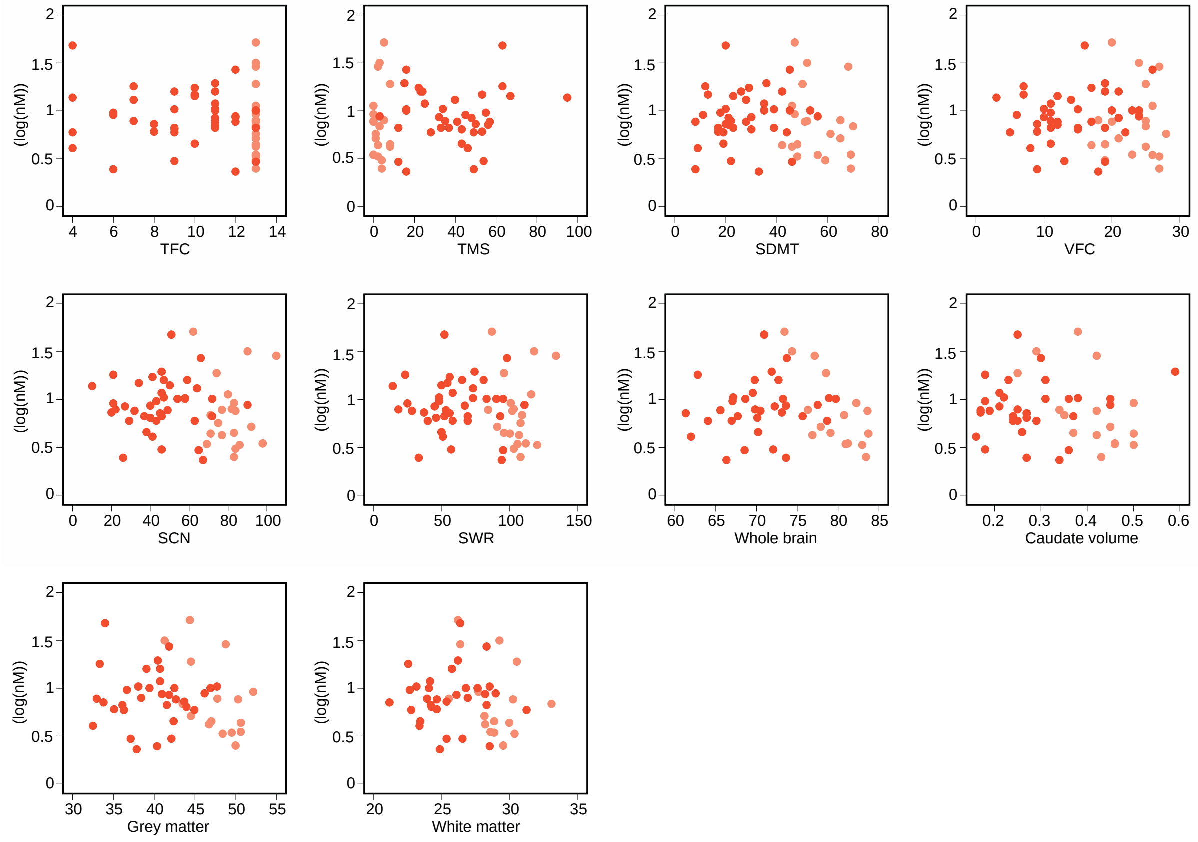

Plasma TRP

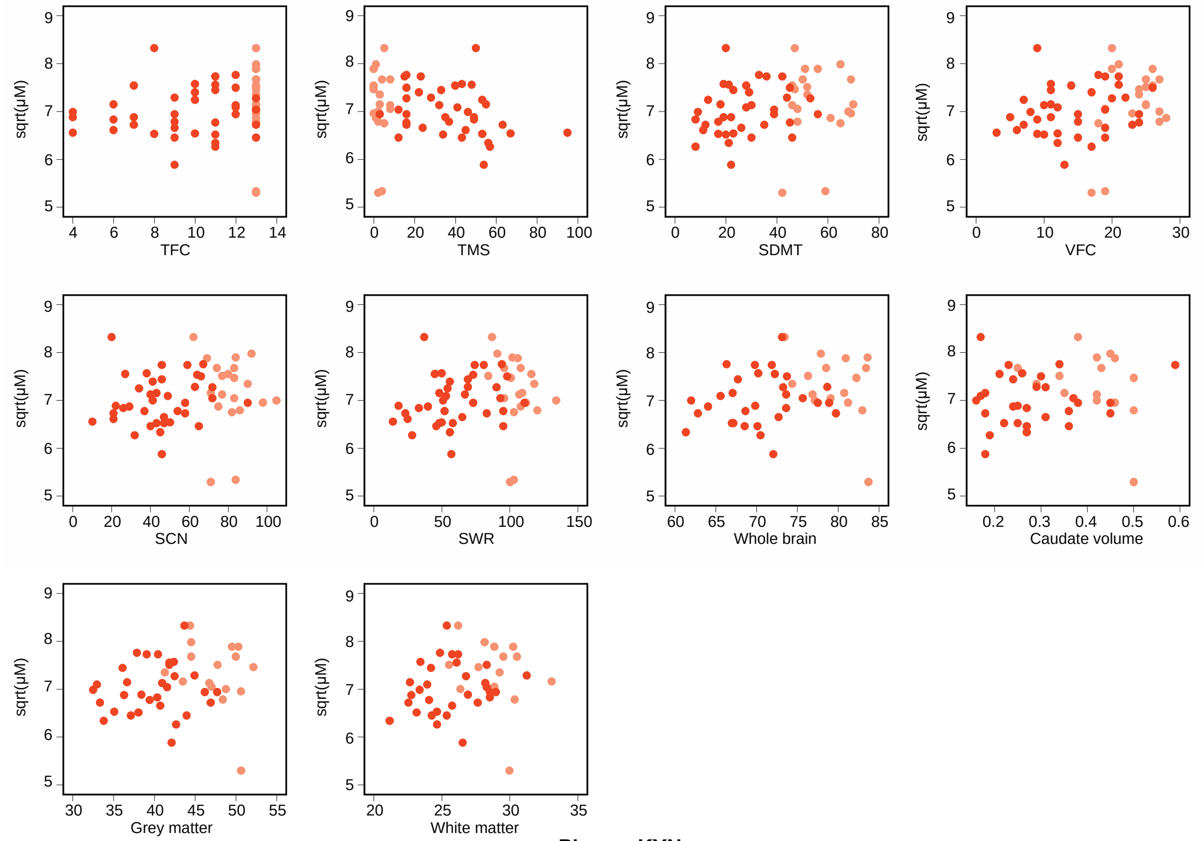

Plasma KYN

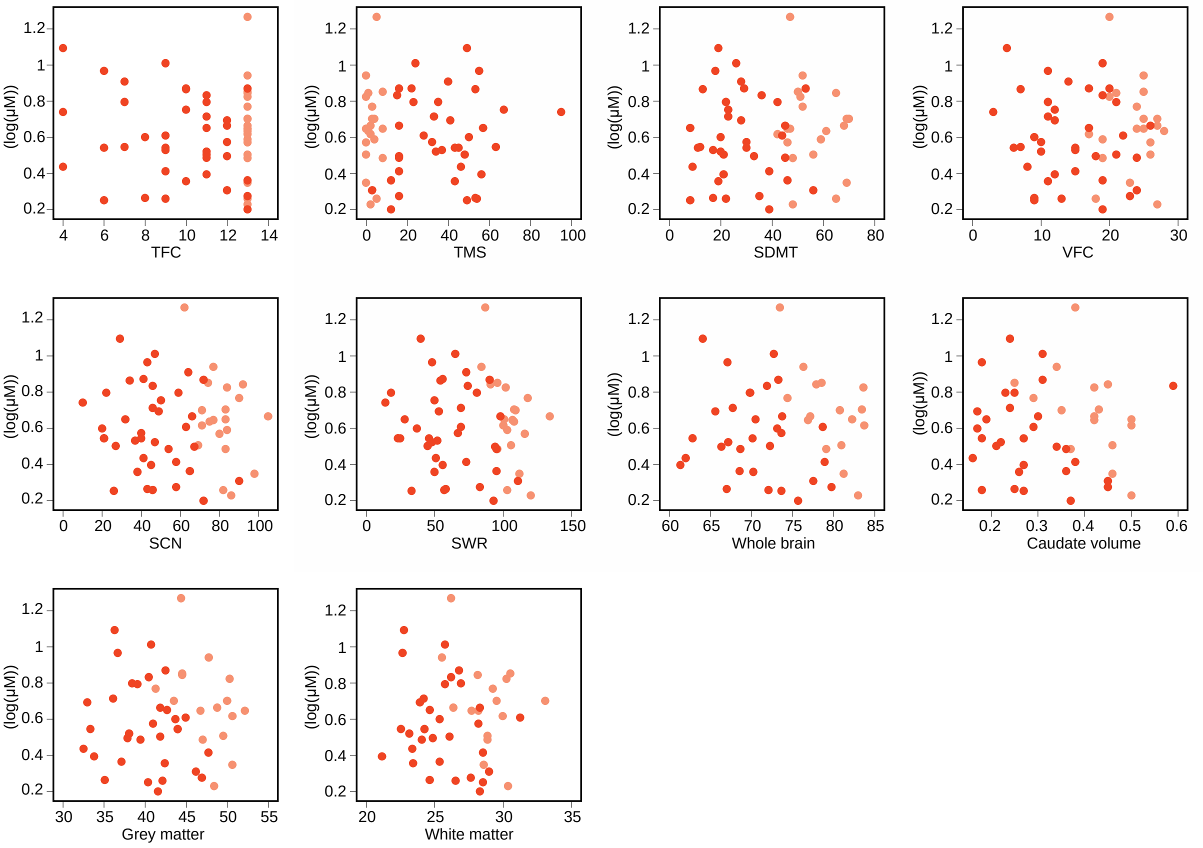

Plasma KYNA

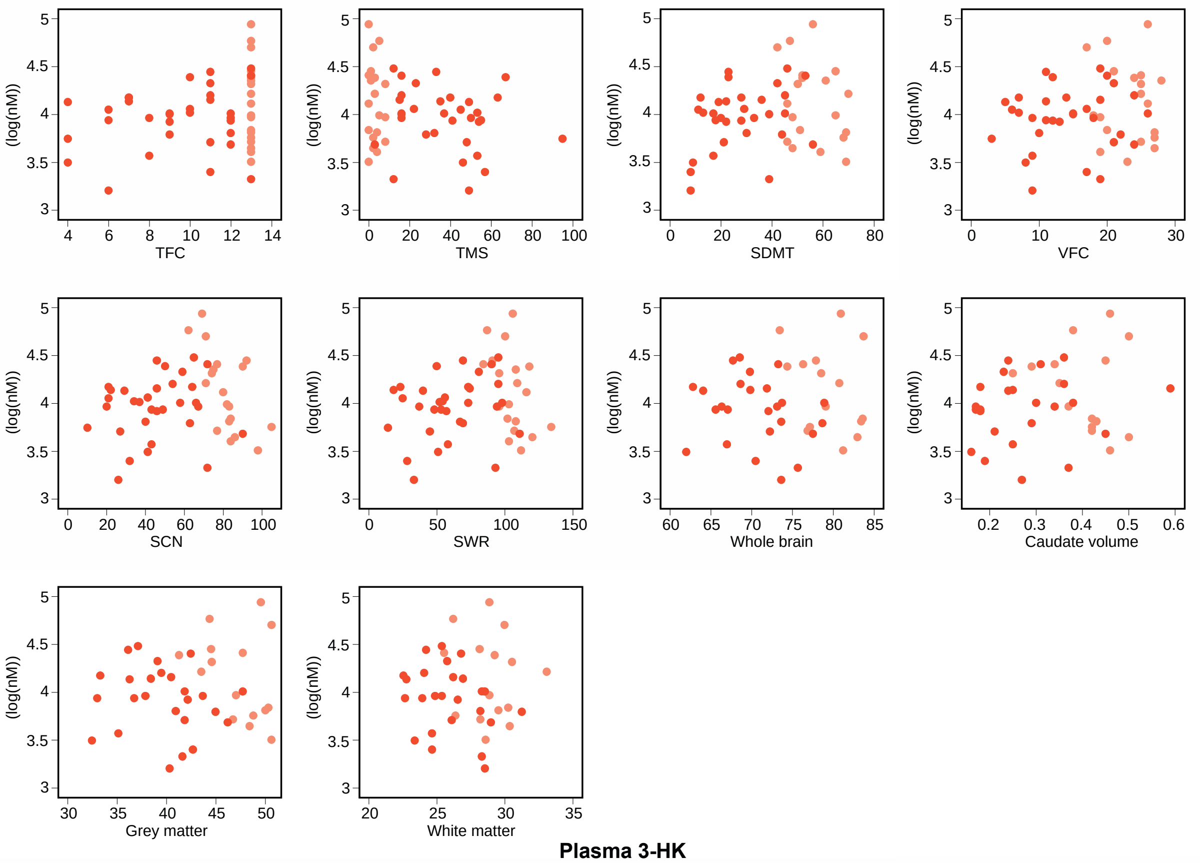

Plasma 3-HK

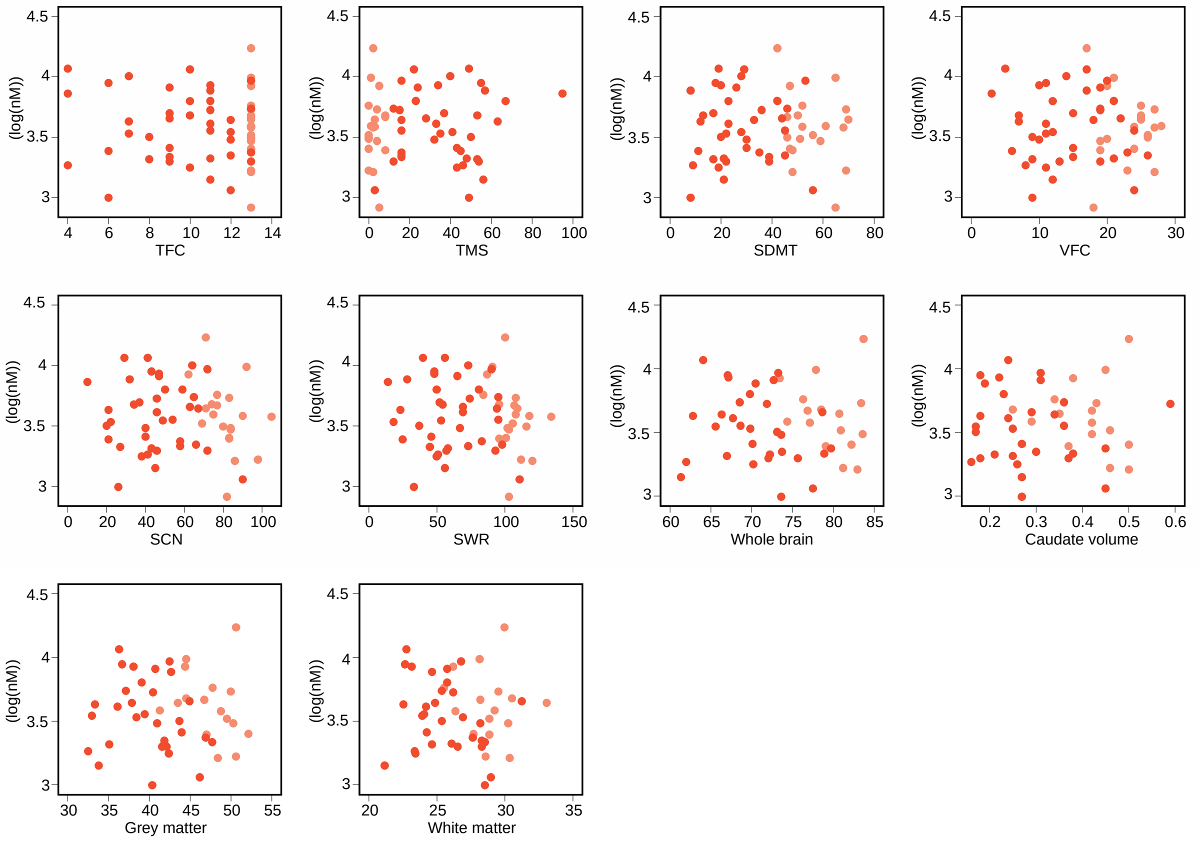

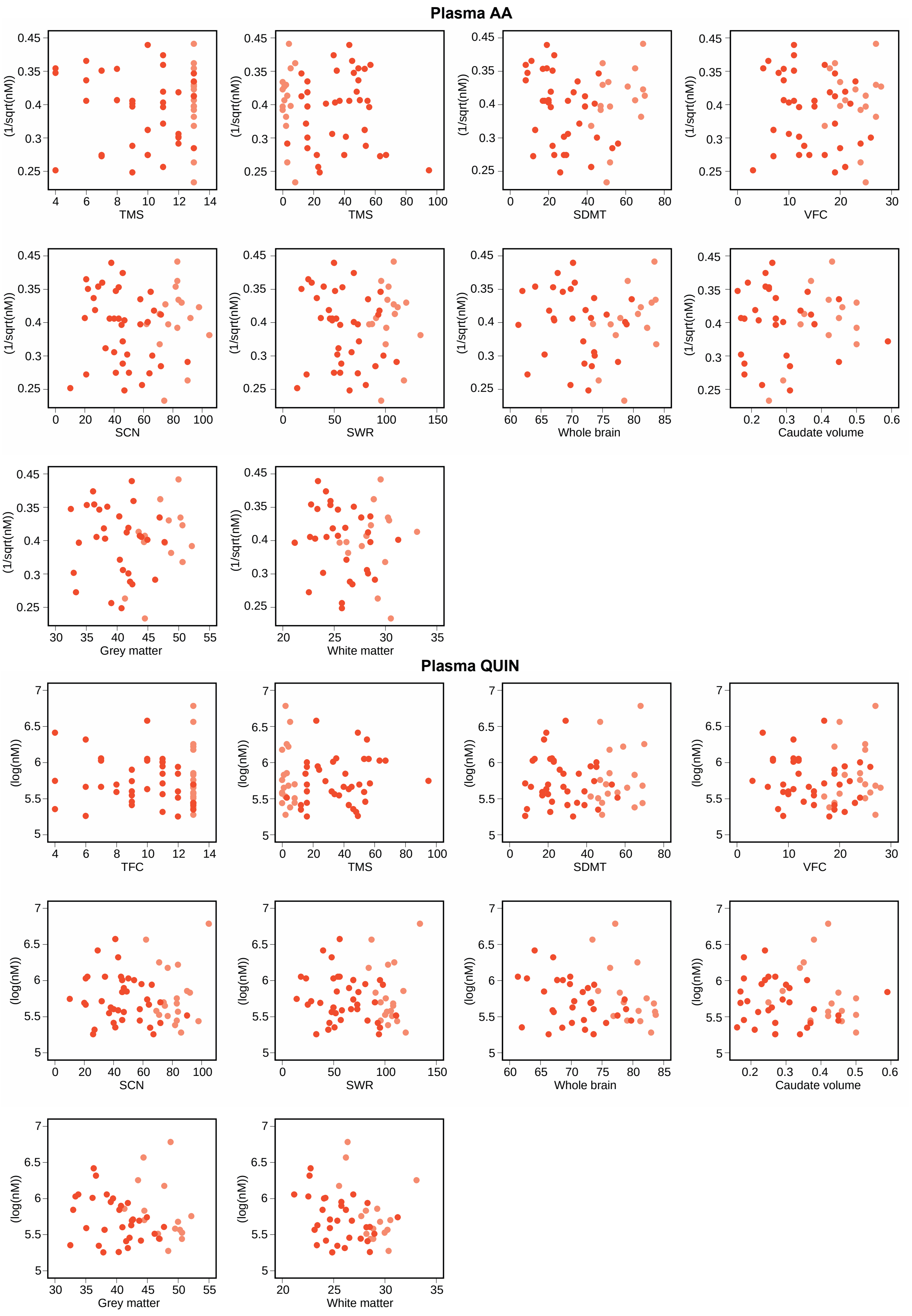

Figure S9 Associations between primary, secondary and exploratory outcomes and clinical and imaging measures. Grey represents healthy controls, light orange preHD and dark orange

HD. 3-HK, 3-hydroxykynurenine; AA, Anthranilic acid; CSF, cerebrospinal fluid; cUHDRS, composite Unified Huntington's Disease Rating Scale; KYN, kynurenine; KYNA, kynurenic acid; QUIN, quinolinic acid; SCN, Stroop Color Naming; SDMT, Symbol Digit Modalities Test; SWR, Stroop Word Reading; TFC, UHDRS Total Functional Capacity; TMS, UHDRS Total Motor Score; TRP, tryptophan; VFC, Verbal Fluency – Categorical.

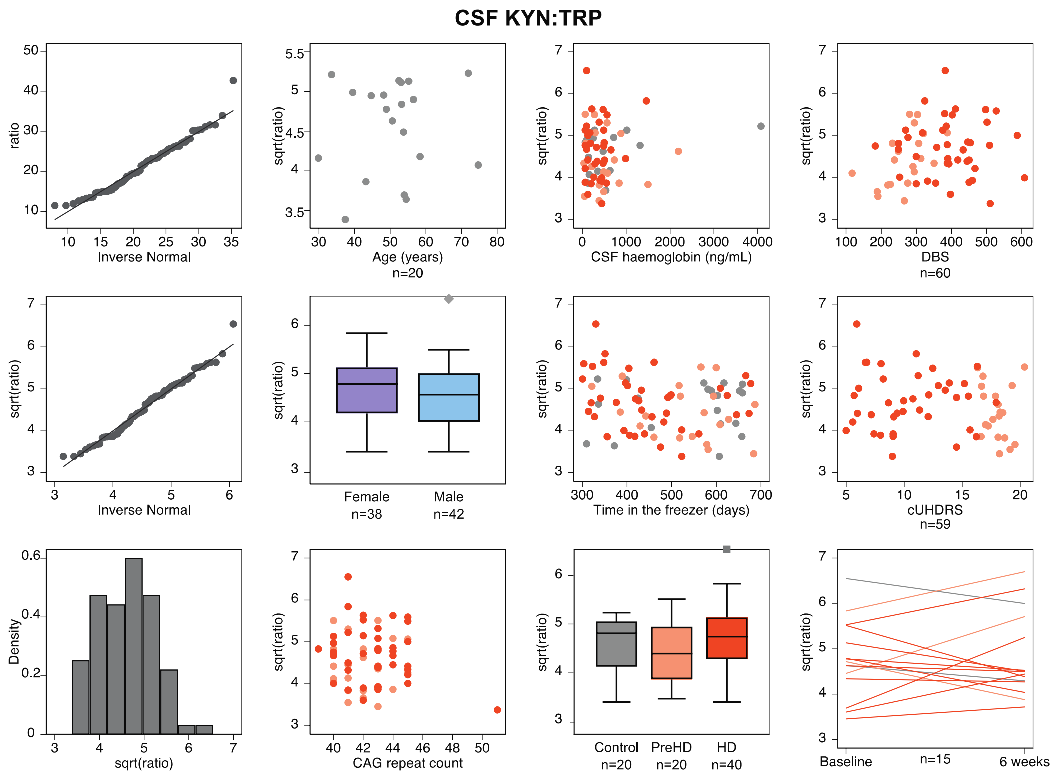

Figure S10 Post-hoc exploratory analyses of CSF KYN:TRP ratio. Distributions and arithmetic operations used (left column): top row depicts a quantile-quantile plot for observed distribution against normal distribution, middle row shows a quantile-quantile plot for transformed distribution against normal distribution, and the bottom row a histogram for transformed distribution. Associations between age (healthy controls only, top row, left middle column), gender (middle row, left middle column), CAG repeat count (gene expansion carriers only, bottom row, left middle column), CSF haemoglobin (top row, right middle column) and time in the freezer (middle row, right middle column). Intergroup differences (bottom row, right middle column), associations with Disease Burden Score (DBS, top row, left column) and composite Unified Huntington's Disease Rating Scale (cUHDRS, middle row, left column), and within-subject short-term stability (bottom row, left column). Associations were not found in any of the analyses. See **Error! Reference source not found.**, Table S2 and Table S3 for point estimates and 95% CI. Grey represents healthy controls, light orange preHD and dark orange HD.

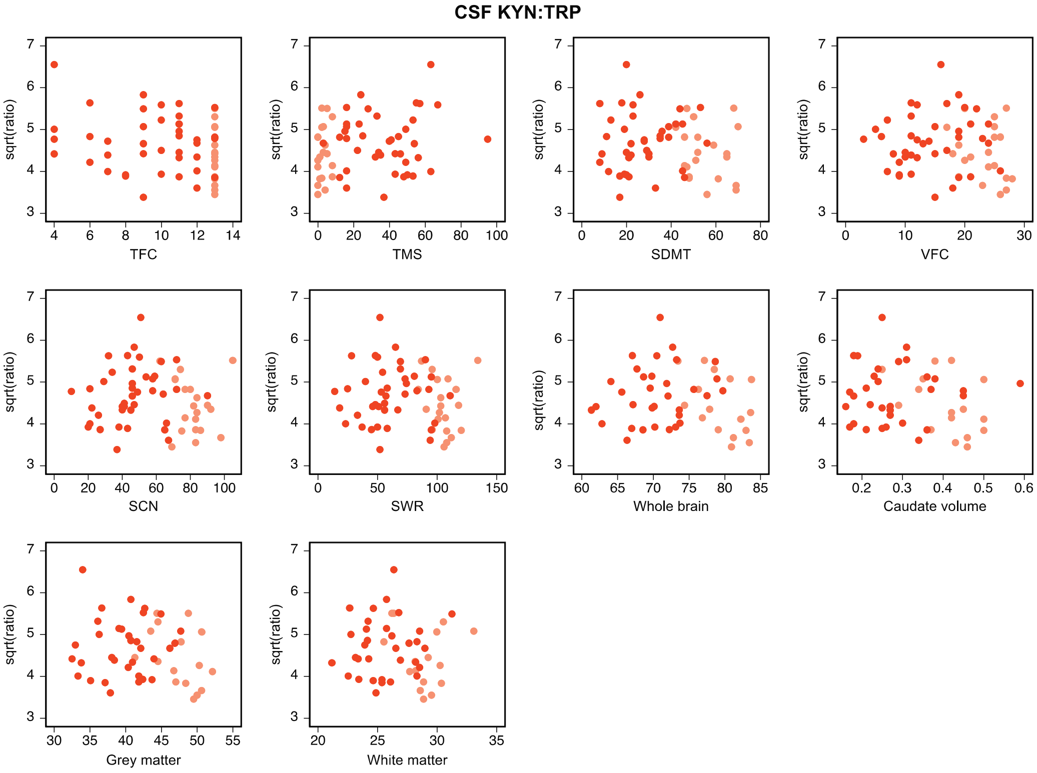

Figure S11 Associations between exploratory post-hoc outcome (CSF KYN:TRP ratio) and clinical and imaging measures. Grey represents healthy controls, light orange preHD and dark orange HD. CSF, cerebrospinal fluid; cUHDRS, composite Unified Huntington's Disease Rating Scale; KYN, kynurenine; SCN, Stroop Color Naming; SDMT, Symbol Digit Modalities Test; SWR, Stroop Word Reading; TFC, UHDRS Total Functional Capacity; TMS, UHDRS Total Motor Score; TRP, tryptophan; VFC, Verbal Fluency – Categorical.
